# Causal Analyses of Associations Between Brain Structure and Suicide Attempt in Adulthood and Late Childhood

**DOI:** 10.1016/j.jaacop.2025.02.005

**Published:** 2025-03-21

**Authors:** Yi Zhou, Luis F.S. Castro-de-Araujo, Madhurbain Singh, Michael C. Neale

**Affiliations:** a Virginia Commonwealth University, Richmond, Virginia

**Keywords:** brain structure, causal analyses, Mendelian randomization, suicide attempt, twin direction-of-causation

## Abstract

**Objective:**

Brain markers for suicide risk in adulthood may be detected during childhood and used for earlier detection and initiation of preventive interventions. Genetic instrumental variable analyses were used to determine whether there is evidence of lower brain total cortical surface area and thinner average cortical thickness (ACT) causing increased suicide risk in adults and whether lower measures of similar brain measures can cause increased risk of suicidality and related psychopathology in older children.

**Method:**

Two-sample Mendelian randomization (MR) was used with summary statistics from genome-wide association studies for total cortical surface area, ACT, and suicide attempt in adults to test causal hypotheses. In youth ages 9 to 10 years old, a combined MR and twin-based direction-of-causation approach was applied to the European twin sample (199 monozygotic, 257 dizygotic twin pairs), and a hybrid traditional twin direction-of-causation approach was applied to the full twin sample (308 monozygotic, 397 dizygotic twin pairs) from the Adolescent Brain Cognitive Development (ABCD) Study.

**Results:**

Two-sample MR analyses found a significant negative causal effect of total cortical surface area on suicide attempt risk in adults. MR–direction-of-causation analyses did not find a significant causal effect of any brain measure on suicidality in older children, but found significant negative causal effects of ACT on depression and internalizing psychopathology, and vice versa.

**Conclusion:**

Brain markers of suicide risk may be instantiated differently in adults compared with older children, though lower ACT may be causally related to psychopathology associated with suicidality in these youth.

## Plain language summary

Using genetic, neuroimaging, and psychiatric measures from adults and older children, we found that lower total brain surface area may be associated with an increased risk of a suicide attempt in adults, but not in older children. Instead, in older children, lower average brain thickness was associated with increased levels of depression and other psychiatric measures known to be associated with suicide risk. These findings suggest that suicide behaviors may be mediated by different brain processes across adults and older children, and thus may require different screening and diagnostic considerations.

Differences in brain structure have been found in both adults and youth who attempt suicide. In a large multicohort study of adult suicide attempt, decreases in total intracranial volume and regional decreases in both surface area and cortical thickness were found in participants who attempted suicide compared with healthy control participants.^1^ In a moderately sized study of adolescents, participants with a history of suicide attempt were also found to have decreased regional surface area in all 17 cortical regions found to be altered compared with healthy control participants.^2^ Although these studies report significant global differences in brain structure associated with suicide attempt, whether altered brain structure causes increased suicide risk remains to be established.

It is also plausible that suicide attempt may alter brain structure. According to the 3-step theory of suicide, for individuals to act on their high levels of suicide desire, the capability for suicide must be present.^3^ The capability for suicide may be acquired through certain life experiences, such as nonsuicidal self-injury and previous suicide attempt, that allow individuals to overcome their fear of suicide death, injury, or pain. Thus, these specific life experiences may lead to changes in the brain to develop the capability for suicide.

In addition to suicide capability, prior psychopathology remains one of the strongest predictors of adult suicide attempt.^4^ Similarly, during childhood and adolescence, substance use, depression, posttraumatic stress disorder, impulsivity, aggression, and exposure to violence remain significant risk factors of suicide behaviors.^5^ Interestingly, both smaller global cortical brain volumes and cortical surface area have been associated with greater general psychopathology in older children.^6^^,^^7^ Another neurobehavioral risk factor implicated in suicide attempt involves alterations in behavioral inhibition system and behavioral activation system (BAS) function which regulate avoidance and approach behaviors, respectively. In a cross-sectional study, concurrent high levels of behavioral inhibition system and BAS sensitivity were found to be positively correlated with risk of past-month suicide attempt risk.^8^ Another study found that elevated BAS fun-seeking scores (a subscale of the BAS measure) were preserved in older children who developed suicide behaviors.^9^

**Figure 1:**
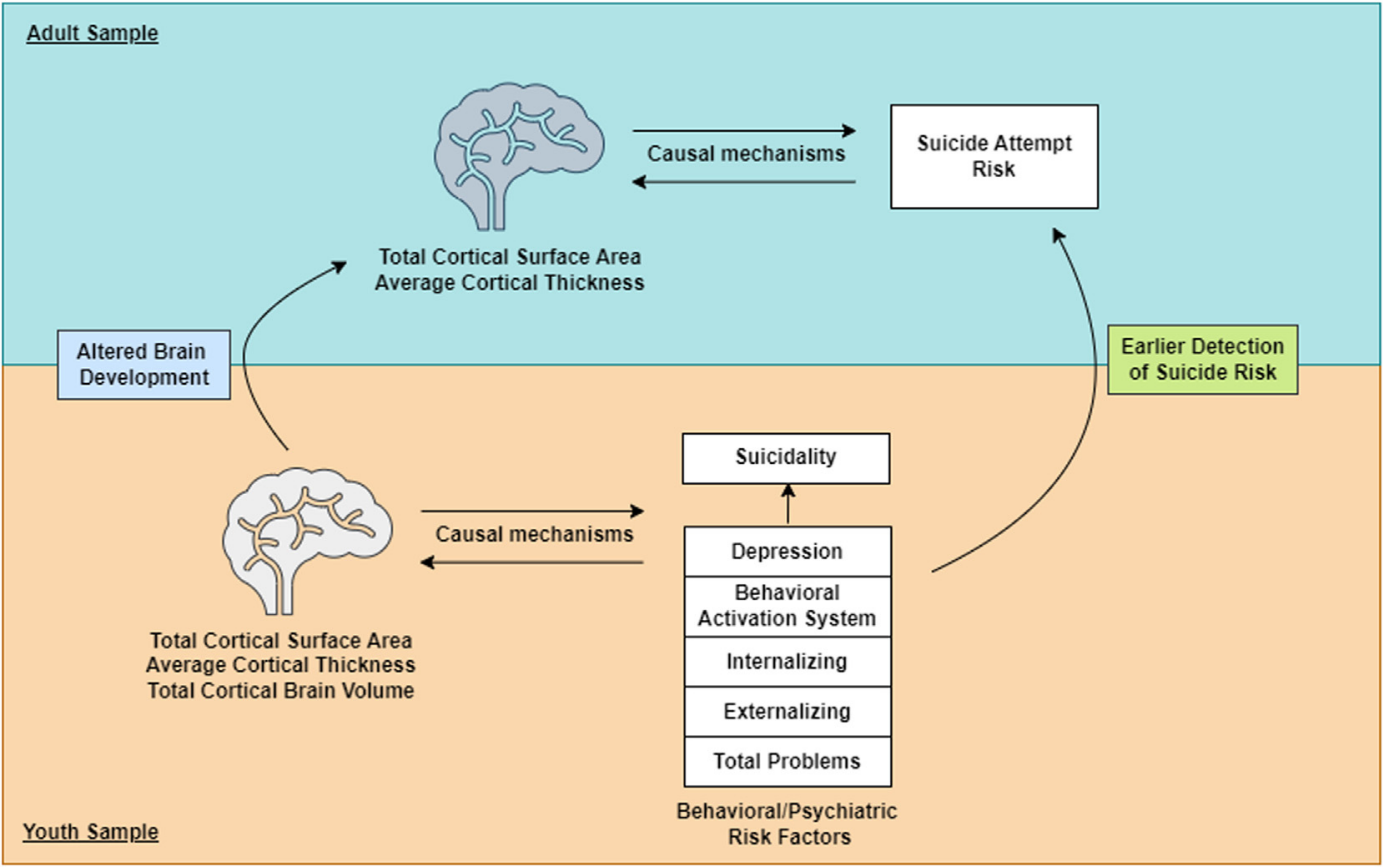
Theoretical Framework Linking Causal Processes Between Altered Brain Structure and Suicide Risk in Older Children and Adults ***Note:****Altered global brain structures in older children may cause increased risk of suicidality or worsened psychopathology leading to suicide attempt later in life. Conversely, behavioral problems and psychopathology during childhood may cause changes in brain structure leading to pathological brain development and altered global brain structure in adults that, in turn, may increase risk of suicide attempt. Of note, it is possible that suicide attempt may also cause changes in global brain structure in adults. Thus, detecting causal processes between brain structure and suicide attempt in adults that may mirror causal processes in late childhood may help identify markers of suicide risk earlier in life.*

Dysregulated brain and behavioral processes found during childhood may contribute to the development of suicide behaviors later in life (Figure 1). As such, any causal processes identified in adults also found in youths are of great importance as they may be used to identify individuals at risk earlier in life.

However, establishing a causal connection between altered brain structure and suicidality remains a practical (and ethical) challenge. Several statistical approaches based on Mendelian randomization (MR) offer avenues to do so using nonexperimental data.^10^^,^^11^ Using summary statistics from genome-wide association studies (GWASs) of the exposure and outcome of interest, a 2-sample MR method can be performed to test for a causal connection between them using genetic instrumental variables (IVs).^10^ Using data from monozygotic (MZ) and dizygotic (DZ) twins, another method that combines MR with twin-based direction-of-causation (MR-DoC) can also be used to assess causality while relaxing some of the assumptions of 2-sample MR.^11^

The first aim of this study was to use 2-sample MR to test the hypothesis that lower brain total cortical surface area (TCSA) and average cortical thickness (ACT) increase the risk of suicide attempt in adults. To determine if the hypothesized causal effects of brain structure on suicidality extend to older children, our second aim was to apply MR-DoC to total cortical brain volume (TCBV), TCSA, ACT, and suicide measures from the European twin sample from the Adolescent Brain Cognitive Development (ABCD) Study. Furthermore, we applied MR-DoC to test our hypotheses that lower global brain structures causally influence depression, BAS fun-seeking, internalizing, externalizing, and total problem scores. Establishing causality is of critical importance because not only does it clarify our understanding of the etiology of suicide behaviors, but it also may help identify more appropriate targets of clinical treatment.

## Method

### Two-Sample Mendelian Randomization

Publicly available GWAS summary statistics were obtained for TCSA and ACT from a study using the UK Biobank sample of 32,488 adults of European ancestry.^12^ The average age of the UK Biobank sample was 64 years and 52% were women.

GWAS summary statistics were also obtained for adult suicide attempt from a multi-ancestry meta-analysis across 22 cohorts including 43,871 cases and 915,025 controls.^13^ Additional details of the meta-analysis cohort characteristics can be found in Supplement 1, available online. Notably, we chose to use 2 GWAS summary statistics derived from meta-analyzed study: one derived from using the European ancestry sample only, which consisted of 35,786 cases and 779, 392 controls, and another derived from using a multi-ancestry sample that included 41,438 cases and 580,259 controls, of which 81% were of European, 11% of African, 5% of East Asian, and 3% of Latin ancestry admixtures. Importantly, the sample used for the multi-ancestry GWAS summary statistics excluded samples from the UK Biobank.

MR uses genetic IVs of an exposure of interest to estimate its causal effect on an outcome (Supplement 1, available online). First, independent single nucleotide polymorphisms (SNPs) significantly associated with the exposure at the genome-wide level from associated GWAS summary statistics were identified. Then, SNPs with corresponding effect estimates in the outcome GWAS were selected as potential genetic IVs. We ensured that SNP outcome effects were not significant at the genome-wide level (ie, *p* > 5 × 10^−8^) (Figure S1, available online). Ambiguous SNPs were excluded, and Steiger filtering was used to assess the validity of the genetic IVs by testing the directionality of the assumed causal effects for each IV.^14^ Exposure and outcome GWAS summary statistics were harmonized by multiplying the SNP effects for the outcome by −1 if the effect and reference alleles were flipped between exposure and outcome summary statistics.

To identify SNPs that may be invalid genetic IVs, we used the GWAS Atlas web tool^15^ to query the SNPs against available GWASs and identified those that were significantly associated (*p* < 5 × 10^−8^) with socioeconomic factors, alcohol use, smoking, and/or educational attainment, similar to the approach used in another MR study.^16^ Additionally, we identified SNPs significantly associated with psychiatric measures, including schizophrenia, as they may be confounders of both brain morphology and suicide risk (Supplement 1, available online). We have taken 2 parallel approaches: a conservative approach in which all SNPs associated with any potential confounders were excluded from subsequent MR analyses and another approach in which all SNPs were kept as genetic IVs and a data-driven method, MR constrained maximum likelihood (MR-cML),^17^ was used to simultaneously estimate causal effects and detect potential invalid IVs using a penalized regression approach (Figure S1, available online). The results were then compared for consistency.

All MR analyses were performed using R version 4.3.1 (R Foundation for Statistical Computing, Vienna, Austria; https://www.r-project.org/) statistical programming language and RStudio version 2023.12.0 (Posit, Boston, Massachusetts; https://posit.co/). For our first more conservative approach whereby SNPs associated with potentially confounding factors were excluded as genetic IVs, we used the MendelianRandomization^18^ package to apply various 2-sample MR approaches, including inverse-variance-weighted (IVW) (random-effects model), weighted median, and MR-Egger^19^ regression methods. Leave-one-out analyses were performed for significant IVW causal effects using the mr_loo function, which sequentially omits one genetic IV at a time and then estimates the IVW causal effect. The impact of individual genetic IVs on the causal estimate may be evaluated this way. We used MR-PRESSO^20^ as an additional sensitivity analysis.

For our second approach, all SNPs were included as genetic IVs, and the MR-cML method was applied using the MRcML^17^ package. The MR-cML method reports results from 2 sub-approaches. One is an Akaike information criterion (AIC)–based approach that generally has greater statistical power but is less stringent in IV selection and thus more susceptible to type I error. The other is a Bayesian information criterion (BIC)–based approach that is more conservative and selective in genetic IVs at the cost of lower statistical power.

Our 2-sample MR analyses were consistent with Strengthening the Reporting of Observational Studies in Epidemiology using MR (STROBE-MR) guidelines.^21^ Checklists can be found in Supplements 2 and 3, available online.

### MR-DoC Modeling

MR-DoC combines an MR approach with traditional twin-based DoC modeling (twin-DoC).^11^ MR-DoC extends the traditional twin-DoC approach by including a polygenic risk score (PRS) of the exposure measure as a genetic IV in the causal model, which allows explicit estimation of the presence of horizontal pleiotropy, a major limitation of classic MR methods (Supplement 1, available online).

#### ABCD Study Twin Sample

The ABCD Study is a national longitudinal study of brain and behavioral development and includes a twin sample of about 379 DZ and 308 MZ twin pairs. Of the twin sample, 66% to 67% identified with White race/ethnicity, and 51% to 52% were male (Table S1, available online). Data from the baseline time point when the study participants were 9 to 10 years of age were used. The data we used were from the ABCD Study 4.0 release (https://doi.org/10.15154/1523041).

#### Measures of Interest

The main structural brain imaging measures of interest were TCBV, ACT, and TCSA. Details of T1- and T2-weighted three-dimensional structural brain image acquisition and processing are described elsewhere.^22^ Briefly, brain cortical surface and subcortical segmentation were performed using FreeSurfer v5.3. Cortical regions were parcellated using Destrieux and Desikan atlases. We used the recommended inclusion criteria for T1-weighted structural neuroimaging measures to include samples that passed imaging quality control.^23^

Responses to self-reported suicidal thoughts and behaviors items were obtained from the Schedule for Affective Disorders and Schizophrenia for School-Age Children (K-SADS). Suicidality was coded as an ordinal measure with 3 levels of increasing suicide liability: no suicidal thoughts and behaviors, suicidal ideation only (past or present passive, active with method, active with intent, active with plan, or nonspecific active ideation, but no suicide behaviors), and suicidal behaviors (past or present preparatory actions toward imminent suicide behaviors and suicide attempt including interrupted or aborted suicide attempt).

For other psychopathology and behavioral measures, we obtained parent-reported measures of their child’s *DSM-5* Depression, Internalizing, Externalizing, and Total Problems scores from the Child Behavior Checklist (CBCL) instrument as well as self-reported BAS fun-seeking scores. Notably, CBCL measures were zero-inflated. To reduce skewness, 1 was added to all responses (to ensure non-zero values) and then subjected to a log10 transformation, a common approach that may be taken.^24^

#### Polygenic Risk Scores

For our MR-DoC analyses, we computed PRSs for brain structure and suicide attempt in the ABCD Study target samples using GWAS summary statistics derived from adults. Notably, applying GWAS summary statistics derived from one ancestry (such as European) to target samples of other ancestries (such as African, East Asian, and Latin) has been shown to lead to unpredictable biases in disease risk in the target sample.^25^^,^^26^ As GWAS summary statistics for brain structure and suicide attempt both were available only from adult samples of European ancestry, we computed PRSs only for brain structure and suicide attempt in ABCD Study samples of European ancestry. Details of PRS computations can be found in Supplement 1, available online.

#### MR-DoC Twin Modeling and Sensitivity Analyses

For PRSs as well as brain and psychiatric/behavioral measures of interest, we regressed out the effects of the top 20 within-ancestry genetic principal components, sex, and interview age as fixed effects. Additionally, we regressed out any confounding effects due to study site as a random effect. The distributions of residuals were then visually inspected to ensure approximate normality.

The umx^27^ and OpenMx^28^ R packages were used to fit MR-DoC models to twin data from the ABCD Study. Specifically, the umxMRDoC function from the umx package was used to fit MR-DoC models. As the measure of suicidality was an ordinal variable, it was analyzed under the liability threshold model,^29^ whereby the observed levels of suicidality were assumed to reflect an underlying continuous liability. The thresholding method used to estimate the deviations was based on the method described by Mehta and Neale,^30^ where the 2 thresholds had fixed values. Note, we assume that there were no unique environmental sources of confounding in our MR-DoC analyses. This assumption is necessary to estimate horizontal pleiotropy and ensure model identification. However, sensitivity analyses were conducted to evaluate how different fixed values of unique environmental confounding impacts the causal estimate (Figure S2B, available online). The *p* values of the causal effect estimates from the MR-DoC models were adjusted for multiple testing by applying Benjamini-Hochberg false discovery rate correction.^31^

#### Hybrid Twin-DoC Model

A hybrid version of the classic twin-DoC approach^32^ that does not require the use of a PRS as a genetic IV was fitted to the entire ABCD Study twin sample to assess the generalizability of significant causal effects identified using MR-DoC to individuals of other ancestries. Additional details can be found in Supplement 1, available online.

## Results

### Two-Sample MR

To test the hypothesis that TCSA causally influences the risk of suicide attempt in adults, we first applied 2-sample MR using summary statistics from GWASs of TCSA and suicide attempt in adults of European ancestry. There were 19 independent SNPs considered as potential genetic IVs. All 19 SNPs passed Steiger filtering, though 1 ambiguous SNP (rs3755019) was removed from subsequent analyses (Table S2, available online). Of the remaining 18 SNPs, 7 were associated with potential confounding factors and thus may be invalid genetic IVs (Tables S2 and S3, available online).

**Figure 2:**
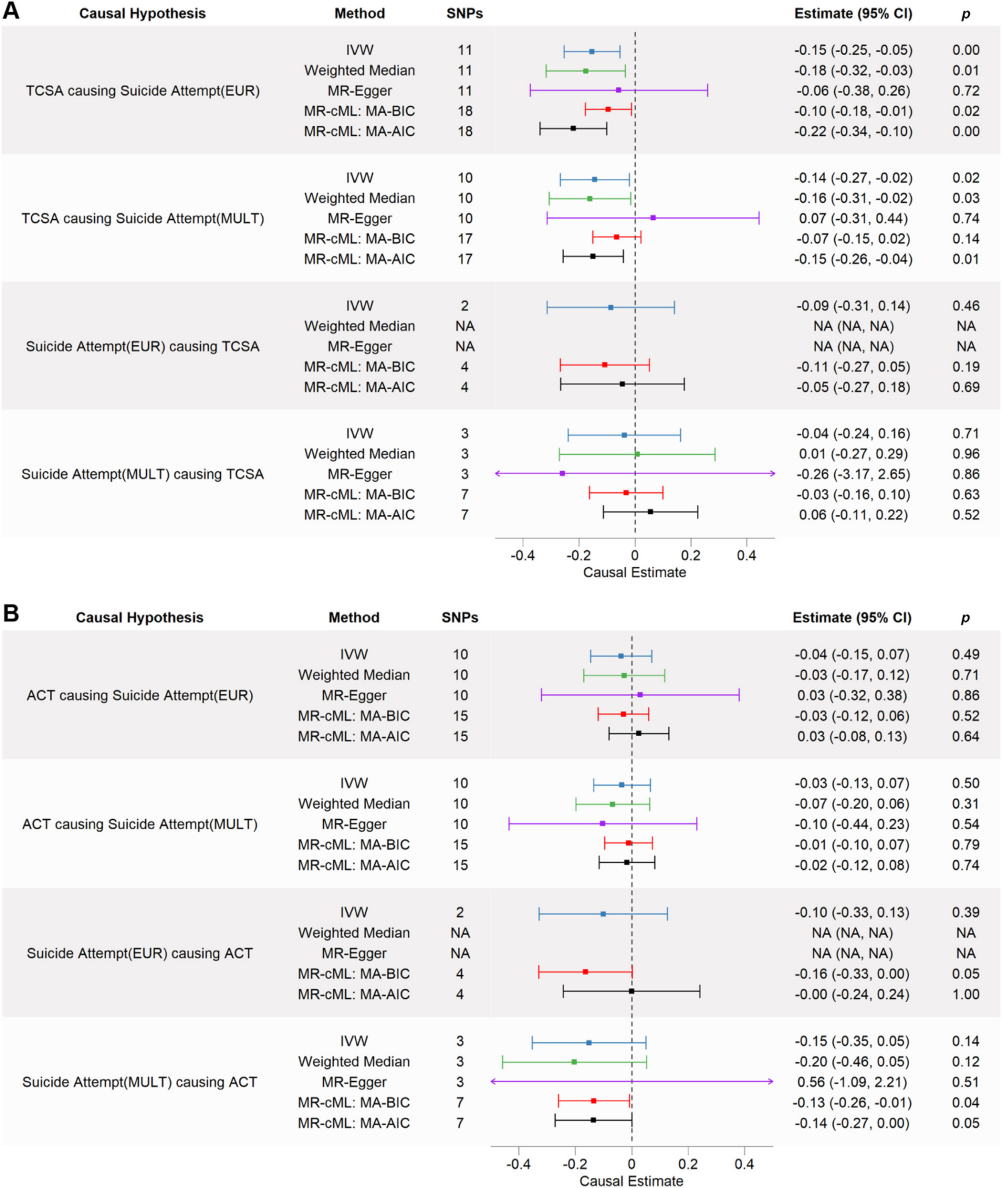
Two-Sample Mendelian Randomization (MR) Estimates of Causal Effects of A) Total Cortical Surface Area (TCSA) and B) Average Cortical Thickness (ACT) on Suicide Attempt Risk ***Note:****The reverse estimated causal effects of suicide attempt on TCSA and ACT are also shown in panels A) and B), respectively. Causal estimates from the inverse-variance-weighted (IVW), weighted-median, MR-Egger, and MR constrained maximum likelihood (MR-cML) approaches are plotted. Error bars represent 95% CIs. Summary statistics for genome-wide association studies of suicide attempt in adults from either a European (EUR) sample or a multi-ancestry (MULT) sample were used. Genome-wide association studies for TCSA and ACT were from adult EUR samples only. MA-AIC = model averaging Akaike information criterion; MA-BIC = model averaging Bayesian information criterion; SNPs = single nucleotide polymorphisms.*

Using the more conservative set of 11 valid genetic IVs (after excluding the 7 potentially invalid genetic IVs), we found a significant negative IVW causal effect of TCSA on suicide attempt (−0.15; 95% CI [−0.25, −0.05]), which remained significant in the weighted-median but not MR-Egger sensitivity analyses (Figure 2A). No single genetic IV driving the causal association was identified with leave-one-out analyses (Figure S3A, available online). No significant global test of heterogeneity was found with MR-PRESSO, and no outliers were detected. As such, the MR-PRESSO causal estimate was equivalent to the IVW estimate (Table S4, available online).

Using the full set of 18 SNPs as genetic IVs, the MR-cML method also found significant negative causal effects in both the AIC-based (−0.22; 95% CI [−0.34, −0.10]) and more conservative BIC-based (−0.10; 95% CI [−0.18, −0.01]) approaches (Figure 2A). The AIC-based approach identified 6 invalid IVs, 4 of which were identified previously as being associated with significant confounding factors, which include schizophrenia/bipolar disorder, education qualifications, and alcohol use (Table S2, available online).

Importantly, participants from the UK Biobank were used in both the GWAS of suicide attempt in adults of European ancestry and the GWAS of TCSA. Thus, sample overlap between these 2 GWASs may contribute to biased causal estimates. However, summary statistics from a GWAS of suicide attempt using a multi-ancestry sample excluding participants from the UK Biobank were available. There were 18 independent SNPs considered as potential genetic IVs. All 18 SNPs passed Steiger filtering, but 1 ambiguous SNP (rs3755019) was excluded from subsequent analyses. Seven SNPs were found to be associated with potential confounding factors, leaving 10 SNPs as a conservative set of valid genetic IVs (Table S2, available online).

Using the more conservative set of 10 genetic IVs, we found a significant negative IVW causal effect of TCSA on suicide attempt risk (−0.14; 95% CI [−0.27, −0.02]), consistent with our earlier results (Figure 2A). However, leave-one-out analyses found 3 SNPs (rs4273712, rs76928645, and rs8756) that may be driving the IVW causal effect (Figure S3B, available online). Significant negative causal effects were found across weighted-median and MR-cML AIC-based, but not BIC-based, methods.

We also assessed the reverse causal hypothesis that suicide attempt causes decreased TCSA. Four independent SNPs significantly associated with suicide attempt from a European ancestry sample GWAS and 8 significant independent SNPs from a multi-ancestry sample GWAS of suicide attempt were considered as potential genetic IVs, though 1 ambiguous SNP from the multi-ancestry GWAS was excluded from subsequent analyses. Notably, 2 SNPS from the European GWAS and 4 SNPs from the multi-ancestry GWAS were associated with potentially confounding factors (Table S2, available online). No significant causal effects were found with any of the MR approaches using the conservative or full set of genetic IVs (Figure 2A).

We repeated the same set of approaches to test the hypothesis that lower ACT causes increased risk of suicide attempt in adults and the reverse causal hypothesis. No significant causal estimates were found in any of the 2-sample MR analyses testing the causal influence of ACT on the risk of suicide attempt (Figure 2B). However, a significant negative causal effect of suicide attempt risk on ACT was found with the MR-cML method using the AIC-based, but not the BIC-based, approach (−0.13; 95% CI [−0.26, −0.01]) (Figure 2B). Though none of the other 2-sample MR analyses evaluating the causal influence of suicide attempt on ACT were significant, few genetic IVs were used in each of those analyses, which may have contributed to low statistical power to detect any true effects.

### MR-DoC Modeling

We applied the MR-DoC method only to the European twin sample from the ABCD Study due to the lack of summary statistics from GWASs of brain structure from other ancestry groups required for computing PRSs used in MR-DoC analyses (see “Method”). At baseline, the ABCD Study participants were 9 to 10 years of age. The ABCD Study European twin sample consisted of about 257 MZ and 199 DZ twin pairs. About 49% of the twin sample were girls, and the average age was approximately 10.2 years. No significant differences were found between MZ and DZ twins in terms of age, sex, brain-based measures, or behavioral/psychiatric measures (Table 1). The prevalence of suicidal ideation was 6% to 7%, and the prevalence of suicide attempt was 1.5%.

**Table 1:** Demographic Characteristics of European Adolescent Brain Cognitive Development (ABCD) Study Twin Sample

Measures	DZ twins, n = 515 (∼257 twin pairs)		MZ twins, n = 398 (∼199 twin pairs)		*p*^a^
	Mean	(SD)	Mean	(SD)	
Interview age, mo	122.2	(6.2)	122.0	(6.5)	.67
	**n**	**(%)**	**n**	**(%)**	
Sex					>.99
Female	248	(49)	193	(49)	
Male	263	(51)	204	(51)	
	**Mean**	**(SD)**	**Mean**	**(SD)**	
TCBV, cm^3^	606	(49)	603	(47)	.3
TCSA, cm^2^	1,904	(158)	1,897	(158)	.5
ACT, mm	2.75	(0.07)	2.75	(0.07)	.36
	**n**	**(%)**	**n**	**(%)**	
Suicidal thoughts and behaviors					.91
No suicidal thoughts and behaviors	470	(92)	362	(91)	
Suicidal ideation only	34	(6.7)	29	(7.3)	
Suicidal behaviors	7	(1.4)	6	(1.5)	
	**Mean**	**(SD)**	**Mean**	**(SD)**	
*DSM-5* Depression residualized scores	−0.04	(0.91)	−0.09	(0.91)	.48
Behavioral Activation System Fun-Seeking residualized scores	0.02	(1.00)	−0.07	(0.92)	.15
Internalizing residualized scores	−0.08	(0.95)	−0.07	(1.04)	.91
Externalizing residualized scores	−0.05	(1.01)	−0.08	(0.98)	.64
Total problems residualized scores	−0.06	(1.03)	−0.11	(1.05)	.51

Note: ACT = average cortical thickness; DZ = dizygotic; MZ = monozygotic; TCBV = total cortical brain volume; TCSA = total cortical surface area.

a Welch 2-sample *t* test (for continuous measures); Pearson χ^2^ test (for categorical measures).

We computed PRSs for TCSA, ACT, and suicide attempt risk in the ABCD Study European sample using summary statistics from GWASs of TCSA, ACT, and suicide attempt in adult samples of European ancestry. Because we did not compute a PRS for TCBV, we used the PRS for TCSA as a genetic IV for both observed measures of TCSA and TCBV. *F* statistics were computed to assess the strength of the PRSs as genetic IVs for the exposures of interest (Table S5, available online). All PRSs exhibited *F* statistics >10, indicating strong genetic IVs, except for the PRS for suicide attempt as a genetic IV for suicidality (*F* = 5.58, *p* = .004) and as a genetic IV for BAS fun-seeking behaviors (*F* = 2.64, *p* = .11). With weak genetic IVs, the MR-DoC model is equivalent to a hybrid version of the traditional twin-DoC model.

**Figure 3:**
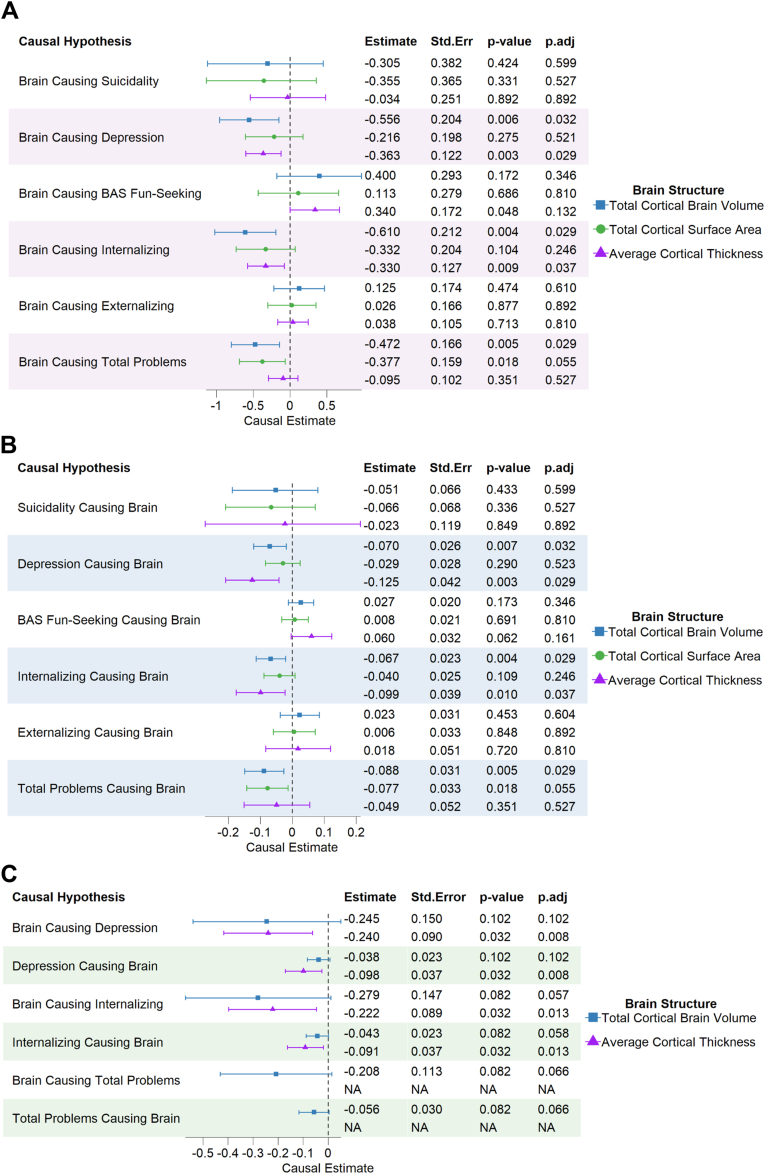
Mendelian Randomization Direction-of-Causation (DoC) and Hybrid Twin-Based DoC Causal Estimates ***Note:****Causal influences A) of total cortical brain volume, total cortical surface area, and average cortical thickness on the risk of suicidality and associated behavioral risk factor measures and B) of the reverse causal influences of suicidality and associated behavioral risk factor measures on total cortical brain volume, total cortical surface area, and average cortical thickness in the European Adolescent Brain Cognitive Development (ABCD) Study twin sample using Mendelian randomization DoC are shown. Significant Mendelian randomization DoC causal estimates were assessed with twin-based DoC analyses using the full ABCD Study twin sample and are shown in C). The* p *values were adjusted (*p_*adj*_*) for multiple testing using Benjamini-Hochberg false discovery rate correction. Error bars represent 95% CIs*. ***BAS =****behavioral activation system*.

In our first set of MR-DoC analyses, we evaluated whether lower TCBV, TCSA, or thinner ACT causes increased suicidality, depression, BAS fun-seeking, internalizing, externalizing, and total problem scores. We did not find significant causal effects of any brain structure measure on suicidality. However, we did find significant negative causal effects of TCBV on depression (−0.56 ± 0.20, adjusted *p* [*p*_adj_] = .03), internalizing (−0.61 ± 0.21, *p*_adj_ = .03), and total problem scores (−0.47 ± 0.17, *p*_adj_ = .03), as well as significant causal effects of ACT on depression (−0.36 ± 0.12, p_adj_ = 0.03) and internalizing (−0.33 ± 0.13, *p*_adj_ = .04) scores (Figure 3A). No significant causal effects were found for TCSA on any of the other psychiatric/behavioral measures.

For our second set of MR-DoC analyses, we tested the reverse causal hypotheses that suicidality and associated psychiatric/behavioral risk factor measures causally influence brain structure. We did not find any significant causal effects of suicidality on any of the brain measures. However, we did find significant negative causal effects of depression on TCBV (−0.07 ± 0.03, *p*_adj_ = .03) and ACT (−0.13 ± 0.04, *p*_adj_ = .03), of internalizing on TCBV (−0.07 ± 0.02, *p*_adj_ = .03) and ACT (−0.10 ± 0.04, *p*_adj_ = .04), and of total problems on TCBV (−0.09 ± 0.03, *p*_adj_ = .03) (Figure 3B).

For MR-DoC analyses with significant causal effect estimates, model estimates of horizontal pleiotropy are reported in Table S6, available online. Estimates of PRS associations with exposures are reported in Table S7, available online.

One major assumption of the MR-DoC model is the absence of unique environmental confounding (covE) or factors confounding the exposure and outcome in one twin of a twin pair, but not in the other twin. To determine how different values of covE would affect the causal estimates, we performed additional sensitivity analyses fixing covE to incrementally smaller and larger values. For all models, larger positive values of covE were associated with more negative causal estimates, whereas more negative covE was associated with more positive causal estimates (Figure S4A-J, available online).

Finally, to assess whether the significant findings in twins of European ancestry may generalize to a more diverse population, we applied the hybrid twin-DoC model, which does not require PRSs as genetic IVs, to the full ABCD Study twin sample. The full twin sample consisted of about 397 DZ and 308 MZ twin pairs, of which about 33% to 34% self-identified as non-White. No differences in demographic, brain, or psychiatric and behavioral measures were found between MZ and DZ twins (Table S1, available online). We found significant negative causal effects of ACT on depression (−0.24 ± 0.09, *p*_adj_ = .03) and internalizing (−0.22 ± 0.09, *p*_adj_ = .03) psychopathology, as well as significant negative reverse causal effects of depression (−0.10 ± 0.04, *p*_adj_ = .03) and internalizing (−0.09 ± 0.04, *p*_adj_ = .03) on ACT (Figure 3C).

## Discussion

Our 2-sample MR analyses using summary statistics from GWASs of adults of European ancestry found evidence suggesting that lower TCSA causes increased suicide attempt risk, but not the reverse. Notably, the MR-cML approach identified several invalid genetic IVs matching those we excluded a priori due to their associations with potential confounding factors, validating our approach. Importantly, we found consistent evidence of a significant negative causal effect of TCSA on suicide attempt risk using summary statistics from a multi-ancestry GWAS of suicide attempt in an adult sample independent from the sample used for the GWAS of TCSA, eliminating any bias due to sample overlap. However, using the multi-ancestry GWAS of suicide attempt may have reduced our statistical power due to differences in linkage disequilibrium structure between populations impacting the strength of genetic associations across different groups.^33^ This may explain why we found a causal effect in our MR-cML AIC-based approach, but not the more conservative BIC-based approach.

Interestingly, using the multi-ancestry GWAS of suicide attempt, we found a significant negative causal effect of suicide attempt on ACT using the MR-cML BIC-based approach only, suggesting that decreased ACT may be a consequence of suicide attempt. No significant causal effects were identified in the IVW and other sensitivity analyses possibly due to low statistical power as few genetic IVs were available for suicide attempt. One interpretation might be that decreases in ACT may underlie increased suicide capability, which may result from prior suicide attempts.^3^ Indeed, 1 study found significantly thinner cortices in the left dorsolateral prefrontal, ventromedial prefrontal, and anterior cingulate cortices in patients at high risk for suicide compared with patients not at high risk.^34^

In older children of European ancestry, we did not find any significant causal effects between global brain structure measures and suicidality. In a previous study of suicidality in older children, only thinner left bank of the superior temporal sulcus was associated with suicidal thoughts and behaviors.^35^ Importantly, we did not distinguish between suicidal ideation and suicidal behaviors due to their low prevalence in the ABCD Study sample at baseline. However, suicidal ideation and suicidal behaviors may be distinct processes^36^ associated with specific neural underpinnings.

However, we did find evidence consistent with TCBV causing increased depression in older children of European ancestry. However, TCBV also exhibited significant negative causal influences on internalizing psychopathology and total problem scores. Furthermore, significant negative reverse causal effects of depression, internalizing, and total problem scores on TCBV were found. However, these effects were not found in the full ABCD Study twin sample using the traditional twin-DoC model, suggesting limited generalizability. We also found significant negative reciprocal causal influences between ACT and depression and ACT and internalizing psychopathology in older children of European ancestry. Importantly, these effects were also found using the traditional twin-DoC method in the full ABCD Study twin sample. As about one-third of the full twin sample includes individuals of non-White race/ethnicity, these findings may be more generalizable across diverse youth populations. Importantly, population stratification due to differences in ancestral genetic background has been shown to confound brain–behavior associations^37^ and is thus a major limitation of our analyses in the European-only twin sample. With greater availability of non-European twin samples and GWASs of brain structure and suicide, future studies may be able to meta-analyze MR-DoC analyses across different ancestry groups to more accurately characterize brain–behavior associations across diverse populations.

Cortical thickness has been shown to decrease in a monotonic linear fashion in most cortical brain regions by 5 years of age,^38^ whereas TCSA and TCBV peak by late childhood or early adolescence before decreasing throughout the rest of adolescence and later adult life.^39^, ^40^, ^41^ Because cortical thickness appears to exhibit significant developmental changes earlier in life, variability in its development may drive much of the psychopathology manifesting earlier in adolescence. Indeed, cortical thinning in the left occipital^42^ and frontal^43^ areas in early adolescence has been found to be associated with major depressive disorder. Conversely, as significant developmental changes in cortical surface area and cortical brain volume appear to begin slightly later in life, they may instantiate the risk of psychopathology and suicide risk in later adolescence and adulthood. Indeed, older adolescents with depression have been found to exhibit significantly lower surface area in frontal, visual, somatosensory, and motor areas.^44^ Thus, using data from the ABCD Study when participants are in mid-to-late adolescence may be more suited to identifying altered TCSA and TCBV markers of suicide risk.

Altogether, our findings suggest that brain markers of suicide risk may be instantiated differently in adulthood compared with late childhood and stress the importance of considering developmental stage when characterizing brain markers of suicide. Differences in brain structure at particular developmental stages may reflect distinct psychopathological processes related to suicide risk, such as perceived burdensomeness, thwarted belongingness, or suicide capability as proposed by the interpersonal theory of suicide.^36^ To date, no objective brain-based clinical tests are used to screen, diagnose, or monitor psychiatric disorders. Thus, further research investigating how altered brain structures impact specific psychopathological processes and vice versa during different developmental stages may lead to clinically meaningful brain markers of suicide risk and its development earlier in life as well as potential targets of intervention.

Finally, we acknowledge several limitations to the approaches we used in this study. Due to our focus on global brain structure measures, we were unable to identify specific functional brain circuits related to suicide risk. Additionally, we did not adjust our brain and behavioral measures for the potential effects of medications or pubertal stage in our MR-DoC analyses, both of which may have a significant impact on brain structure and behavior.^45^^,^^46^ Furthermore, we adjusted brain and behavioral measures for sex by regressing out its effects, which meant that we could not estimate any sex differences in our analyses. Future studies examining the causal connection between specific neurocircuits and behavioral risk factors may further elucidate the mechanisms driving suicide behaviors. Furthermore, accounting for the potential confounding effects of psychotropic medications^47^ and further investigations into how sex-based differences in brain structure and suicide behavior epidemiology^48^ might be related to altered neurocircuit development will lead to a clearer understanding of the nuanced mechanisms underlying suicidal behaviors in older children and adults.

## CRediT authorship contribution statement

**Yi Zhou:** Writing – review & editing, Writing – original draft, Methodology, Investigation, Funding acquisition, Formal analysis, Conceptualization. **Luis F.S. Castro-de-Araujo:** Writing – review & editing, Software, Methodology, Investigation, Conceptualization, Supervision. **Madhurbain Singh:** Writing – review & editing, Methodology, Conceptualization, Investigation. **Michael C. Neale:** Writing – review & editing, Supervision, Methodology, Conceptualization, Funding acquisition, Investigation, Resources, Software.

## References

[1] Campos A.I., Thompson P.M., Veltman D.J. (2021). Brain correlates of suicide attempt in 18,925 participants across 18 international cohorts. Biol Psychiatry.

[2] Gifuni A.J., Chakravarty M.M., Lepage M. (2021). Brain cortical and subcortical morphology in adolescents with depression and a history of suicide attempt. J Psychiatry Neurosci.

[3] Klonsky E.D., Pachkowski M.C., Shahnaz A., May A.M. (2021). The three-step theory of suicide: description, evidence, and some useful points of clarification. Prev Med.

[4] Franklin J.C., Ribeiro J.D., Fox K.R. (2017). Risk factors for suicidal thoughts and behaviors: a meta-analysis of 50 years of research. Psychol Bull.

[5] Hink A.B., Killings X., Bhatt A., Ridings L.E., Andrews A.L. (2022). Adolescent suicide—understanding unique risks and opportunities for trauma centers to recognize, intervene, and prevent a leading cause of death. Curr Trauma Rep.

[6] Romer A.L., Ren B., Pizzagalli D.A. (2023). Brain structure relations with psychopathology trajectories in the ABCD Study. J Am Acad Child Adolesc Psychiatry.

[7] Durham E.L., Jeong H.J., Moore T.M. (2021). Association of gray matter volumes with general and specific dimensions of psychopathology in children. Neuropsychopharmacology.

[8] Bryan C.J., Kyron M., Page A.C. (2022). BIS sensitivity, BAS sensitivity, and recent suicide attempts. Pers Individ Dif.

[9] Zhou Y., Neale M. (Posted online February 9, 2024). Adolescent suicide behaviors are associated with accelerated reductions in cortical gray matter volume and maintenance of behavioral activation system sensitivity. Research Square.

[10] Burgess S., Davey Smith G., Davies N.M. (2023). Guidelines for performing Mendelian randomization investigations: update for summer 2023. Wellcome Open Res.

[11] Minică C.C., Dolan C.V., Boomsma D.I., de Geus E., Neale M.C. (2018). Extending causality tests with genetic instruments: an integration of mendelian randomization with the classical twin design. Behav Genet.

[12] Makowski C., van der Meer D., Dong W. (2022). Discovery of genomic loci of the human cerebral cortex using genetically informed brain atlases. Science.

[13] Docherty A.R., Mullins N., Ashley-Koch A.E. (2023). GWAS meta-analysis of suicide attempt: identification of 12 genome-wide significant loci and implication of genetic risks for specific health factors. Am J Psychiatry.

[14] Hemani G., Tilling K., Smith G.D. (2017). Orienting the causal relationship between imprecisely measured traits using GWAS summary data. PLoS Genet.

[15] Watanabe K., Stringer S., Frei O. (2019). A global overview of pleiotropy and genetic architecture in complex traits. Nat Genet.

[16] Guo J., Yu K., Dong S.S. (2022). Mendelian randomization analyses support causal relationships between brain imaging-derived phenotypes and risk of psychiatric disorders. Nat Neurosci.

[17] Xue H., Shen X., Pan W. (2021). Constrained maximum likelihood-based Mendelian randomization robust to both correlated and uncorrelated pleiotropic effects. Am J Hum Genet.

[18] Patel A., Ye T., Xue H. (2023). MendelianRandomization v0.9.0: updates to an R package for performing Mendelian randomization analyses using summarized data. Wellcome Open Res.

[19] Burgess S., Thompson S.G. (2017). Interpreting findings from Mendelian randomization using the MR-Egger method. Eur J Epidemiol.

[20] Verbanck M., Chen C.Y., Neale B., Do R. (2018). Detection of widespread horizontal pleiotropy in causal relationships inferred from Mendelian randomization between complex traits and diseases. Nat Genet.

[21] Skrivankova V.W., Richmond R.C., Woolf B.A.R. (2021). Strengthening the Reporting of Observational Studies in Epidemiology using Mendelian Randomisation (STROBE-MR): explanation and elaboration. BMJ.

[22] Hagler D.J., Hatton S., Cornejo M.D. (2019). Image processing and analysis methods for the Adolescent Brain Cognitive Development Study. Neuroimage.

[23] Casey B.J., Cannonier T., Conley M.I. (2018). The Adolescent Brain Cognitive Development (ABCD) Study: imaging acquisition across 21 sites. Dev Cogn Neurosci.

[24] West R.M. (2022). Best practice in statistics: The use of log transformation. Ann Clin Biochem.

[25] Kim M.S., Patel K.P., Teng A.K., Berens A.J., Lachance J. (2018). Genetic disease risks can be misestimated across global populations. Genome Biol.

[26] Martin A.R., Gignoux C.R., Walters R.K. (2017). Human demographic history impacts genetic risk prediction across diverse populations. Am J Hum Genet.

[27] Bates T.C., Maes H., Neale M.C. (2019). umx: twin and path-based structural equation modeling in R. Twin Res Hum Genet.

[28] Neale M.C., Hunter M.D., Pritikin J.N. (2016). OpenMx 2.0: extended structural equation and statistical modeling. Psychometrika.

[29] Verhulst B., Neale M.C. (2021). Best practices for binary and ordinal data analyses. Behav Genet.

[30] Mehta P.D., Neale M.C., Flay B.R. (2004). Squeezing interval change from ordinal panel data: latent growth curves with ordinal outcomes. Psychol Methods.

[31] Benjamini Y., Hochberg Y. (1995). Controlling the false discovery rate—a practical and powerful approach to multiple testing. J R Statist Soc B.

[32] Maes H.H., Neale M.C., Kirkpatrick R.M., Kendler K.S. (2021). Using multimodel inference/model averaging to model causes of covariation between variables in twins. Behav Genet.

[33] Gurdasani D., Barroso I., Zeggini E., Sandhu M.S. (2019). Genomics of disease risk in globally diverse populations. Nat Rev Genet.

[34] Wagner G., Schultz C.C., Koch K., Schachtzabel C., Sauer H., Schlösser R.G. (2012). Prefrontal cortical thickness in depressed patients with high-risk for suicidal behavior. J Psychiatr Res.

[35] Vidal-Ribas P., Janiri D., Doucet G.E. (2021). Multimodal neuroimaging of suicidal thoughts and behaviors in a US population-based sample of school-aged children. Am J Psychiatry.

[36] Van Orden K.A., Merrill K.A., Joiner T.E. (2005). Interpersonal-psychological precursors to suicidal behavior: a theory of attempted and completed suicide. Curr Psychiatry Rev.

[37] Huang T.H., Loughnan R., Thompson W.K., Fan C.C. (Posted online August 11, 2022). Preprint.

[38] Ducharme S., Albaugh M.D., Nguyen T.V. (2016). Trajectories of cortical thickness maturation in normal brain development—the importance of quality control procedures. Neuroimage.

[39] Hedman A.M., van Haren N.E.M., Schnack H.G., Kahn R.S., Hulshoff Pol H.E. (2012). Human brain changes across the life span: a review of 56 longitudinal magnetic resonance imaging studies. Hum Brain Mapp.

[40] Wierenga L.M., Langen M., Oranje B., Durston S. (2014). Unique developmental trajectories of cortical thickness and surface area. Neuroimage.

[41] Lenroot R.K., Giedd J.N. (2006). Brain development in children and adolescents: insights from anatomical magnetic resonance imaging. Neurosci Biobehav Rev.

[42] Kim J.H., Suh S.I., Lee H.J., Lee J.H., Lee M.S. (2019). Cortical and subcortical gray matter alterations in first-episode drug-naïve adolescents with major depressive disorder. Neuroreport.

[43] Bos M.G.N., Peters S., van de Kamp F.C., Crone E.A., Tamnes C.K. (2018). Emerging depression in adolescence coincides with accelerated frontal cortical thinning. J Child Psychol Psychiatry.

[44] Schmaal L., Hibar D.P., Sämann P.G. (2017). Cortical abnormalities in adults and adolescents with major depression based on brain scans from 20 cohorts worldwide in the ENIGMA Major Depressive Disorder Working Group. Mol Psychiatry.

[45] Herting M.M., Sowell E.R. (2017). Puberty and structural brain development in humans. Front Neuroendocrinol.

[46] Singh M.K., Chang K.D. (2012). The neural effects of psychotropic medications in children and adolescents. Child Adolesc Psychiatr Clin N Am.

[47] Andersen S.L., Navalta C.P. (2004). Altering the course of neurodevelopment: a framework for understanding the enduring effects of psychotropic drugs. Int J Dev Neurosci.

[48] Kotila L., Lönnqvist J. (1988). Adolescent suicide attempts: sex differences predicting suicide. Acta Psychiatr Scand.

