# Supplementary material for: Causal Analyses of Associations Between Brain Structure and Suicide Attempt in Adulthood and Late Childhood
Source: JAACAP Open. 2025 Mar 21;3(3):455–66. doi: 10.1016/j.jaacop.2025.02.005 (PMC12414325; doi:10.1016/j.jaacop.2025.02.005)
Supplement: Supplementary Material [file mmc1.docx]

Supplement 1

**Supplementary Methods**

**A. Two-Sample Mendelian Randomization (MR)**

MR approaches have been described as being analogous to “nature’s randomized controlled trial” as they rely on the random segregation and independent assortment of germline genetic variants strongly associated with exposures of interest, which are then used as instrumental variables (IVs) to test for, and estimate, the putative causal effects of the exposure on an outcome of interest^6^. If summary statistics from genome-wide association studies (GWAS) of the exposure and outcome of interest are available, then two-sample MR can be performed to test for consistency with a causal relationship between them^1^.

While MR is a powerful tool to probe causality, it relies on several strong assumptions, including that genetic IVs are strongly associated with the exposure of interest (relevance assumption), are not associated with any confounders of the exposure and outcome (independence assumption), and only associated with the outcome through the exposure (the exclusion restriction assumption), and not through alternative mechanisms, which would be considered horizontal pleiotropic associations^3^.

*A.1 Identification of SNPs associated with Confounders as Invalid IVs*

SNPs associated with factors that may confound an exposure (ex. brain structure) and outcome (ex. suicide risk) of interest may be invalid genetic IVs and may be excluded from MR analyses. For example, another MR study found evidence for a positive causal effect of schizophrenia (SZN) on higher brain volume and surface area in the pars-orbitalis region^4^. As SZN is also a risk factor for suicide, SZN may independently impact both suicide risk and brain structure, thus confounding any direct causal relationship between the latter two. However, it is also possible that SZN and altered brain morphology may exist along the same causal pathway impacting suicide risk, and thus considered a type of vertical pleiotropy that would not violate the assumptions of MR. To account for both scenarios, we took two separate approaches where in one, we excluded SNPs associated with potential confounders from being genetic IVs in our analyses, and another where we included all SNPs associated with the exposure and used a data-driven approach called MR-cML^5^ to identify invalid genetic IVs and estimate the causal effect simultaneously **(Figure S1)**.

*A.2 Cohort Characteristics from Multi-Ancestry Meta-Analysis GWAS of Suicide Attempt*

This multi-cohort study combined two large sub-studies which included the International Suicide Genetics Consortium (ISGC) sample of 29,782 cases and 519,961 controls of predominantly European ancestry^6^, and the Million Veterans Program (MVP) sample of 14,089 cases and 395,064 controls of veterans from the USA^7^. The cases in the MVP sample were on average 52 years of age, 85% male, and 65% of EUR ancestry while the controls were on average 63 years of age, 92% male, and 73% of EUR ancestry.

**B. Mendelian Randomization Direction of Causation (MR-DoC) Modeling**

The traditional twin direction-of-causation (twin-DoC) approach leverages differences in the expected cross-twin cross-trait covariances that would arise when different causal hypotheses are specified between two traits (e.g., X causes Y, or Y causes X), to evaluate evidence for causality in a particular direction by comparing model fits^8,9^.

By using a polygenic risk score (PRS) of an exposure phenotype as an IV, MR-DoC extends the traditional twin-DoC approach by explicitly modeling any horizontal pleiotropic associations between the PRS and the outcome measure, in addition to estimating the exposure’s causal effect on the outcome, thereby relaxing the no horizontal pleiotropy assumption **(Figure S2A)**. MR-DoC thus overcomes a significant limitation of the classical MR approach where the presence of any horizontal pleiotropy would render the genetic IV invalid.

Notably, MR-DoC models assume unidirectional causation and do not explicitly test for bi-directionality. To address this issue, an extension of MR-DoC, called MR-DoC2, has been developed, which is capable of explicitly modeling bi-directional causation^39^.

*B.1 Computing Polygenic Risk Scores (PRS)*

To assign genetic ancestries to ABCD study participants, their genotypic data were obtained using the Affymetrix Axiom Smokescreen Genotyping Array, as described elsewhere^10^. We performed genetic ancestry assignment following a previously published protocol, with the 1000 Genomes Project (1KGP) Phase 3^11^ as the external reference panel^12^. Briefly, the 1KGP data comprises 26 “populations” from five continental “super-populations”: Admixed American, African, East Asian, European, and South Asian^11^. SNPs shared between the 1KGP and the ABCD study samples were identified and LD pruned using PLINK 1.9^13^. We then performed principal components analysis (PCA) with the smartPCA function from the EIGENSOFT package^14^, using the 1KGP data to compute the first 10 genetic principal components (PCs) and their corresponding SNP weights. The ABCD samples were then projected onto this PC space based on the SNP weights. Using the 10 PCs, we calculated every ABCD sample’s Mahalanobis distance from the median of each reference “population” and then assigned the sample to the population with the minimum Mahalanobis distance. The corresponding “super-population” was considered to be the assigned genetic ancestry. Within each assigned ancestry group, the Mahalanobis distances were rescaled to have median = 0 and S.D. = 1, and the samples with Mahalanobis distance >3 S.D. were identified as ancestry outliers and removed from subsequent analyses.

For ABCD study participants of EUR ancestry, we used imputed genotypic data and performed QC by removing SNPs with MAF < 0.01, exhibiting significant Hardy Weinberg Disequilibrium (p < 1e-6), or with a missing genotype rate of >1%, and excluding individuals with a missing genotype rate of >1%. After obtaining GWAS summary statistics, we performed QC by removing SNPs with minor allele frequencies (MAF) < 0.01, those with INFO scores < 0.8, ambiguous SNPs (A/T or G/C variants), and duplicate SNPs.

To compute the PRSs, we used PRS-CS^15^ to generate posterior SNP effect estimates using the QC’d GWAS summary statistics and an external European LD reference panel constructed from the UK Biobank data. We then used PLINK’s (v1.9) --score command to compute individual-level PRSs using the posterior SNP effect estimates and the QC’d target ABCD study imputed genotype data.

PRSs are known to be confounded by the effects of population stratification. As such, adjusting PRSs by genetic principal components (PCs), even within relatively more homogenous populations, is often necessary. To compute within-ancestry genetic PCs for participants of European ancestry from the ABCD study, we used PC-AiR^16^, a statistical method able to generate principal components unconfounded by relatedness in the sample. 32 principal components were generated. These principal components can be regressed out from measures of interest, or controlled for as covariates, in subsequent statistical analyses such as MR-DoC.

*B.2 Hybrid Twin-Direction of Causation (twin-DoC) Models*

While a methodological comparison of MR-DoC and the hybrid twin-DoC approaches is out of the scope of this study, both approaches estimate a causal effect of an exposure on an outcome.

Similar to the measures used in MR-DoC, we adjusted brain structural and psychiatric/behavioral measures for the effects of population stratification, sex, and age at baseline by regressing out the effects of genetic principal components derived from the entire ABCD sample (pre-computed and available in the ABCD 4.0 release), sex, and age as fixed effects, and study site as a random effect. We then used the *OpenMx* R package to fit our hybrid twin-DoC models (See supplementary code). Similar to MR-DoC, the hybrid twin-DoC models assume no unique sources of environmental confounding, Forward and reverse causal estimates between brain and psychiatric behavioral measures were then obtained.

**References**

1. Burgess, S. *et al.* Guidelines for performing Mendelian randomization investigations: update for summer 2023. *Wellcome Open Res.* **4**, 186 (2023).

2. Sanderson, E. *et al.* Mendelian randomization. *Nat. Rev. Methods Primer* **2**, 1–21 (2022).

3. de Leeuw, C., Savage, J., Bucur, I. G., Heskes, T. & Posthuma, D. Understanding the assumptions underlying Mendelian randomization. *Eur. J. Hum. Genet.* **30**, 653–660 (2022).

4. Guo, J. *et al.* Mendelian randomization analyses support causal relationships between brain imaging-derived phenotypes and risk of psychiatric disorders. *Nat. Neurosci.* **25**, 1519–1527 (2022).

5. Xue, H., Shen, X. & Pan, W. Constrained maximum likelihood-based Mendelian randomization robust to both correlated and uncorrelated pleiotropic effects. *Am. J. Hum. Genet.* **108**, 1251–1269 (2021).

6. Mullins, N. *et al.* Dissecting the Shared Genetic Architecture of Suicide Attempt, Psychiatric Disorders, and Known Risk Factors. *Biol. Psychiatry* **91**, 313 (2021).

7. Kimbrel, N. A. *et al.* A Genome-Wide Association Study of Suicide Attempts in the Million Veterans Program Identifies Evidence of Pan-Ancestry and Ancestry-Specific Risk Loci. *Mol. Psychiatry* **27**, 2264 (2022).

8. McAdams, T. A., Rijsdijk, F. V., Zavos, H. M. S. & Pingault, J.-B. Twins and Causal Inference: Leveraging Nature’s Experiment. *Cold Spring Harb. Perspect. Med.* **11**, a039552 (2021).

9. Verhulst, B. & Estabrook, R. Using genetic information to test causal relationships in cross-sectional data. *J. Theor. Polit.* **24**, 328–344 (2012).

10. Fan, C. C., Loughnan, R., Wilson, S. & Hewitt, J. K. Genotype data and derived genetic instruments of Adolescent Brain Cognitive Development Study® for better understanding of human brain development. *Behav. Genet.* **53**, 159–168 (2023).

11. 1000 Genomes Project Consortium *et al.* A global reference for human genetic variation. *Nature* **526**, 68–74 (2015).

12. Peterson, R. E. *et al.* The utility of empirically assigning ancestry groups in cross-population genetic studies of addiction. *Am. J. Addict.* **26**, 494–501 (2017).

13. Purcell, S. *et al.* PLINK: A Tool Set for Whole-Genome Association and Population-Based Linkage Analyses. *Am. J. Hum. Genet.* **81**, 559–575 (2007).

14. Patterson, N., Price, A. L. & Reich, D. Population Structure and Eigenanalysis. *PLoS Genet.* **2**, e190 (2006).

15. Ge, T., Chen, C.-Y., Ni, Y., Feng, Y.-C. A. & Smoller, J. W. Polygenic prediction via Bayesian regression and continuous shrinkage priors. *Nat. Commun.* **10**, 1776 (2019).

16. Conomos, M. P., Miller, M. B. & Thornton, T. A. Robust inference of population structure for ancestry prediction and correction of stratification in the presence of relatedness. *Genet. Epidemiol.* **39**, 276–293 (2015).
