# Supplementary material for: Causal Analyses of Associations Between Brain Structure and Suicide Attempt in Adulthood and Late Childhood
Source: JAACAP Open. 2025 Mar 21;3(3):455–66. doi: 10.1016/j.jaacop.2025.02.005 (PMC12414325; doi:10.1016/j.jaacop.2025.02.005)
Supplement: Supplementary Material [file mmc2.docx]

**STROBE-MR checklist of recommended items to address in reports of Mendelian randomization studies**^1^ ^2^**– Two-sample MR**

| **Item No.** | **Section** | **Checklist item** | **Page No.** | **Relevant text from manuscript** |
| --- | --- | --- | --- | --- |
| 1 | **TITLE and ABSTRACT** | Indicate Mendelian randomization (MR) as the study’s design in the title and/or the abstract if that is a main purpose of the study | 1 | “Using genetic instrumental variable analyses, this study aimed to determine whether there is evidence of lower brain total cortical surface area (TCSA) and thinner average cortical thickness (ACT) causing increased suicide attempt risk in adults.” |
|  | **INTRODUCTION** |  |  |  |
| 2 | **Background** | Explain the scientific background and rationale for the reported study. What is the exposure? Is a potential causal relationship between exposure and outcome plausible? Justify why MR is a helpful method to address the study question | 2 | “However, establishing a causal relationship between altered brain structure and suicidality remains a practical (and ethical) challenge. Fortunately, several statistical approaches based on Mendelian Randomization (MR) offer avenues to do so using non-experimental data^10,11^. If summary statistics from genome-wide association studies (GWAS) of the exposure and outcome of interest are available, then the two-sample MR method can be performed to test for a causal relationship between them using genetic instrumental variables (IVs)^10^” |
| 3 | **Objectives** | State specific objectives clearly, including pre-specified causal hypotheses (if any). State that MR is a method that, under specific assumptions, intends to estimate causal effects | 4 | “The first aim of this study was to use two-sample MR to test the hypothesis that lower brain total cortical surface area (TCSA) and average cortical thickness (ACT) increases the risk of suicide attempt in adults.” |
|  | **METHODS** |  |  |  |
| 4 | **Study design and data sources** | Present key elements of the study design early in the article. Consider including a table listing sources of data for all phases of the study. For each data source contributing to the analysis, describe the following: |  |  |
|  | a) | Setting: Describe the study design and the underlying population, if possible. Describe the setting, locations, and relevant dates, including periods of recruitment, exposure, follow-up, and data collection, when available. | 5 | “Publicly available GWAS summary statistics were obtained for TCSA and ACT from a study using the UK Biobank (UKB) sample of 32,488 adults of EUR ancestry^12^. The average age of the UKB sample was 64 years and 52% were female.  GWAS summary statistics were also obtained for adult suicide attempt from a multi-ancestry meta-analysis across 22 cohorts including 43,871 cases and 915,025 controls^13^. Additional details of the meta-analysis cohort characteristics can be found in the **Supplementary Methods A.2.”** |
|  | b) | Participants: Give the eligibility criteria, and the sources and methods of selection of participants. Report the sample size, and whether any power or sample size calculations were carried out prior to the main analysis | NA | Reported in the GWAS studies from which we derived summary statistics. |
|  | c) | Describe measurement, quality control and selection of genetic variants | 6  STable1 | “independent SNPs significantly associated with the exposure at the genome-wide level from associated GWAS summary statistics were identified. Then, SNPs with corresponding effect estimates in the outcome GWAS were then selected as potential genetic IVs. Note, we ensured SNP-outcome effects were not significant at the genome-wide level (ie., p>5e-8) **(Supplementary Figure 1)**.”  “Ambiguous SNPs were excluded and Steiger filtering was used to assess the validity of the genetic IVs by testing the directionality of the assumed causal effects for each IV^14^.”  “In step 1, plausible genetic instrumental variables (IV) were first selected by finding Single Nucleotide Polymorphisms (SNPs) significantly associated with the exposure of interest. Importantly, only linkage disequilibrium (LD)-independent SNPs (such as those identified by using PLINK^20^ to perform LD pruning with the following parameters: LD R^2^ = 0.1, 250k base-pair windows) were selected. In step 2, matching SNPs with corresponding association estimates for the outcome measure were identified.” |
|  | d) | For each exposure, outcome, and other relevant variables, describe methods of assessment and diagnostic criteria for diseases | NA | Reported in the GWAS studies from which we derived summary statistics. |
|  | e) | Provide details of ethics committee approval and participant informed consent, if relevant | 25 | “This study was approved by the Institutional Review Board at Virginia Commonwealth University which deemed it qualified for exemption (HM20025460) according to 45 CFR 46 category 4 (ii): Secondary research for which consent is not required when information is recorded in such a manner that the identity of the subjects cannot readily be ascertained, the investigator does not contact the subjects, and will not re-identify subjects.” |
| 5 | **Assumptions** | Explicitly state the three core IV assumptions for the main analysis (relevance, independence and exclusion restriction) as well assumptions for any additional or sensitivity analysis | Supplementary Methods | Main Analyses:  “While MR is a powerful tool to probe causality, it relies on several strong assumptions, including that genetic IVs are strongly associated with the exposure of interest (relevance assumption), are not associated with any confounders of the exposure and outcome (independence assumption), and only associated with the outcome through the exposure (the exclusion restriction assumption), and not through alternative mechanisms, which would be considered horizontal pleiotropic associations^3^” |
| 6 | **Statistical methods: main analysis** | Describe statistical methods and statistics used |  |  |
|  | a) | Describe how quantitative variables were handled in the analyses (i.e., scale, units, model) | NA | Described in the original research articles the GWAS summary statistics were derived from. |
|  | b) | Describe how genetic variants were handled in the analyses and, if applicable, how their weights were selected | 6 | “Exposure and outcome GWAS summary statistics were harmonized by multiplying the SNP effects for the outcome by -1 if the effect and reference alleles were flipped between exposure and outcome summary statistics.” |
|  | c) | Describe the MR estimator (e.g. two-stage least squares, Wald ratio) and related statistics. Detail the included covariates and, in case of two-sample MR, whether the same covariate set was used for adjustment in the two samples | 7 | “we used the *MendelianRandomization*^18^ package to apply various two-sample MR approaches, including Inverse-Variance-Weighted (IVW) (random-effects model), Weighted-Median, and MR-Egger ^19^ regression methods. Leave-one-out (LOO) analyses were performed for significant IVW causal effects using the *mr_loo() function* which sequentially omits one genetic IV at time and then estimates the IVW causal effect*.* The impact of individual genetic IVs on the causal estimate may be evaluated this way. Furthermore, we utilized MR-PRESSO^20^ as an additional sensitivity analysis.” |
|  | d) | Explain how missing data were addressed | NA | Reported in the GWAS studies from which we derived summary statistics. |
|  | e) | If applicable, indicate how multiple testing was addressed | NA | NA |
| 7 | **Assessment of assumptions** | Describe any methods or prior knowledge used to assess the assumptions or justify their validity | 6 | “To identify SNPs that may be invalid genetic IVs, we used the GWAS-Atlas web-tool^15^ to query the SNPs against available GWASs and identified those that were significantly associated (p<5e-8) with socioeconomic factors, alcohol use, smoking, and/or educational attainment, similar to the approach used in another MR study^16^. Additionally, we identified SNPs significantly associated with psychiatric measures, including Schizophrenia (SZN), as they may be confounders of both brain morphology and suicide risk **(see Supplementary Methods A.1 for additional details)**.” |
| 8 | **Sensitivity analyses and additional analyses** | Describe any sensitivity analyses or additional analyses performed (e.g. comparison of effect estimates from different approaches, independent replication, bias analytic techniques, validation of instruments, simulations) | 7 | “For our second approach, all SNPs were included as genetic IVs and the cML-MA method was applied using the *MRcML*^21^ package.” |
| 9 | **Software and pre-registration** |  |  |  |
|  | a) | Name statistical software and package(s), including version and settings used | 7 | “All MR analyses were performed using the R (version 4.3.1) statistical programming language and R-Studio (version 2023.12.0). We used the MendelianRandomization18 package to apply various two-sample MR approaches” |
|  | b) | State whether the study protocol and details were pre-registered (as well as when and where) | NA | This study was not pre-registered. |
|  | **RESULTS** |  |  |  |
| 10 | **Descriptive data** |  |  |  |
|  | a) | Report the numbers of individuals at each stage of included studies and reasons for exclusion. Consider use of a flow diagram | NA | NA for two-sample MR using summary statistics. |
|  | b) | Report summary statistics for phenotypic exposure(s), outcome(s), and other relevant variables (e.g. means, SDs, proportions) | NA | Reported in the GWAS studies from which we derived summary statistics. |
|  | c) | If the data sources include meta-analyses of previous studies, provide the assessments of heterogeneity across these studies | NA | NA for two-sample MR using summary statistics. |
|  | d) | For two-sample MR:  i.  Provide justification of the similarity of the genetic variant-exposure associations between the exposure and outcome samples  ii.  Provide information on the number of individuals who overlap between the exposure and outcome studies | i) NA  ii) 5 | i) NA for two-sample MR using summary statistics.  ii) “Notably, we chose to use two GWAS summary statistics derived from meta-analyzed study: 1) one derived from using the EUR ancestry sample only, which consisted of 35,786 cases and 779, 392 controls and 2) another derived from using a multi-ancestry sample which included 41,438 cases and 580,259 controls, of which roughly 81% were of EUR, 11% of African (AFR), 5% of East Asian (EAS), and 3% of Latin (LAT) ancestry admixtures. Importantly, the sample used for the multi-ancestry GWAS summary statistics excluded samples from the UKB.” |
| 11 | **Main results** |  |  |  |
|  | a) | Report the associations between genetic variant and exposure, and between genetic variant and outcome, preferably on an interpretable scale | NA | Effect sizes may require permissions from corresponding authors of the respective GWAS research articles. |
|  | b) | Report MR estimates of the relationship between exposure and outcome, and the measures of uncertainty from the MR analysis, on an interpretable scale, such as odds ratio or relative risk per SD difference | Figure 2 | See Figure 2. |
|  | c) | If relevant, consider translating estimates of relative risk into absolute risk for a meaningful time period | NA | NA for our analyses. |
|  | d) | Consider plots to visualize results (e.g. forest plot, scatterplot of associations between genetic variants and outcome versus between genetic variants and exposure) | Figure 2 | See Figure 2. |
| 12 | **Assessment of assumptions** |  |  |  |
|  | a) | Report the assessment of the validity of the assumptions | STable 5, STable 2, STable 4  STable 7 | Relevance Assumption: See F-statistics in Supplementary Table 5 and variant-exposure associations in Supplementary Table 7.  Independence Assumption: See supplementary Table 2 for associations between SNPs and potential confounders.  Restriction Exclusion Assumption: See Supplementary Table for MR-PRESSO statistics assessing the presence of heterogeneity. |
|  | b) | Report any additional statistics (e.g., assessments of heterogeneity across genetic variants, such as *I^2^*, Q statistic or E-value) | NA | NA |
| 13 | **Sensitivity analyses and additional analyses** |  |  |  |
|  | a) | Report any sensitivity analyses to assess the robustness of the main results to violations of the assumptions | Figure 2 | See Figure 2 for results form Weighted-Median, MR-Egger, MR-PRSESO, and MR-cML. |
|  | b) | Report results from other sensitivity analyses or additional analyses | 7 | “For our second approach, all SNPs were included as genetic IVs and the cML-MA method was applied using the *MRcML*^21^ package. The MR-cML method reports results from two sub-approaches. One is an Akaike Information Criterion (AIC)-based approach that generally has greater statistical power but is less stringent in IV selection and thus more susceptible to type I error. The other is a Bayesian Information Criterion (BIC)-based approach that is more conservative and selective in genetic IVs at the cost of lower statistical power. “ |
|  | c) | Report any assessment of direction of causal relationship (e.g., bidirectional MR) | 12 | “We also assessed the reverse causal hypothesis that suicide attempt causes decreased TCSA. 4 independent SNPs significantly associated with suicide attempt from a EUR ancestry sample GWAS and 8 significant independent SNPs from a multi-ancestry sample GWAS of suicide attempt were considered as potential genetic IVs, though one ambiguous SNP from the multi-ancestry GWAS was excluded form subsequent analyses.” |
|  | d) | When relevant, report and compare with estimates from non-MR analyses | NA | NA for our analyses. |
|  | e) | Consider additional plots to visualize results (e.g., leave-one-out analyses) | SF3 | See Supplementary Figure 3. |
|  | **DISCUSSION** |  |  |  |
| 14 | **Key results** | Summarize key results with reference to study objectives | 15 | “Our two-sample MR analyses using summary statistics from GWAS of adults of EUR ancestry found evidence suggesting that lower TCSA causes increased suicide attempt risk, but not the other way around.” |
| 15 | **Limitations** | Discuss limitations of the study, taking into account the validity of the IV assumptions, other sources of potential bias, and imprecision. Discuss both direction and magnitude of any potential bias and any efforts to address them | 16 | “However, using the mutli-ancestry GWAS of suicide attempt may have reduced our statistical power due to differences in Linkage Disequilibrium (LD) structure between populations that may affect the strength of genetic associations across different groups^34^. This may explain why only our MR-cML AIC-based approach, but not the more conservative BIC-based approach, found a significant causal effect.”  “No significant causal effects were identified in the IVW and other sensitivity analyses possibly due to low statistical power as few genetic IVs were available for suicide attempt.” |
| 16 | **Interpretation** |  |  |  |
|  | a) | Meaning: Give a cautious overall interpretation of results in the context of their limitations and in comparison with other studies | 18 | “Altogether, our findings suggest that suicide risk may be instantiated differently in adults compared to youth. As such, clinical interventions aimed at reducing the rate by which cortical surface area decreases in later adolescence may more effectively decrease the risk of suicide in adults.” |
|  | b) | Mechanism: Discuss underlying biological mechanisms that could drive a potential causal relationship between the investigated exposure and the outcome, and whether the gene-environment equivalence assumption is reasonable. Use causal language carefully, clarifying that IV estimates may provide causal effects only under certain assumptions | 16 | “One interpretation might be that decreases in ACT may underlie increased suicide capability, which may result from prior suicide attempts, or other painful and provocative events^3^.” |
|  | c) | Clinical relevance: Discuss whether the results have clinical or public policy relevance, and to what extent they inform effect sizes of possible interventions | 18 | “Altogether, our findings suggest that suicide risk may be instantiated differently in adults compared to youth. As such, clinical interventions aimed at reducing the rate by which cortical surface area decreases in later adolescence may more effectively decrease the risk of suicide in adults.” |
| 17 | **Generalizability** | Discuss the generalizability of the study results (a) to other populations, (b) across other exposure periods/timings, and (c) across other levels of exposure | 16 | “However, using the mutli-ancestry GWAS of suicide attempt may have reduced our statistical power due to differences in Linkage Disequilibrium (LD) structure between populations that may affect the strength of genetic associations across different groups^34^. This may explain why only our MR-cML AIC-based approach, but not the more conservative BIC-based approach, found a significant causal effect.” |
|  | **OTHER INFORMATION** |  |  |  |
| 18 | **Funding** | Describe sources of funding and the role of funders in the present study and, if applicable, sources of funding for the databases and original study or studies on which the present study is based | Title page |  |
| 19 | **Data and data sharing** | Provide the data used to perform all analyses or report where and how the data can be accessed, and reference these sources in the article. Provide the statistical code needed to reproduce the results in the article, or report whether the code is publicly accessible and if so, where | 26, 27 | “GWAS summary statistics for total brain cortical surface area9 and suicide attempt10 were obtained from publicly available repositories.  Data used in the preparation of this article were obtained from the Adolescent Brain Cognitive DevelopmentSM (ABCD) Study (https://abcdstudy.org), held in the NIMH Data Archive (NDA)…”  “The scripts used for genetic ancestry assignment and genetic principal component analysis are available upon request. Scripts for all the other analyses supporting this study are available at our Open Science Framework repository for this study: https://osf.io/xe8yp/?view_only=3daf23ae35e94c1d92d8d31326ca4a1d” |
| 20 | **Conflicts of Interest** | All authors should declare all potential conflicts of interest | 27 | “The author(s) declare no competing interests.” |

This checklist is copyrighted by the Equator Network under the Creative Commons Attribution 3.0 Unported (CC BY 3.0) license.

1. Skrivankova VW, Richmond RC, Woolf BAR, Yarmolinsky J, Davies NM, Swanson SA, et al. Strengthening the Reporting of Observational Studies in Epidemiology using Mendelian Randomization (STROBE-MR) Statement. JAMA. 2021;under review.

2. Skrivankova VW, Richmond RC, Woolf BAR, Davies NM, Swanson SA, VanderWeele TJ, et al. Strengthening the Reporting of Observational Studies in Epidemiology using Mendelian Randomisation (STROBE-MR): Explanation and Elaboration. BMJ. 2021;375:n2233.
