# Supplementary material for: Causal Analyses of Associations Between Brain Structure and Suicide Attempt in Adulthood and Late Childhood
Source: JAACAP Open. 2025 Mar 21;3(3):455–66. doi: 10.1016/j.jaacop.2025.02.005 (PMC12414325; doi:10.1016/j.jaacop.2025.02.005)
Supplement: Supplementary Material [file mmc3.docx]

**STROBE-MR checklist of recommended items to address in reports of Mendelian randomization studies**^1^ ^2^**– MR-DoC**

| **Item No.** | **Section** | **Checklist item** | **Page No.** | **Relevant text from manuscript** |
| --- | --- | --- | --- | --- |
| 1 | **TITLE and ABSTRACT** | Indicate Mendelian randomization (MR) as the study’s design in the title and/or the abstract if that is a main purpose of the study | 1 | “We applied two-sample Mendelian Randomization (MR) using publicly available Genome-Wide-Association-Study summary statistics for total brain cortical surface area and suicide attempt in adults to test whether the data are consistency with a causal relationship.” |
|  | **INTRODUCTION** |  |  |  |
| 2 | **Background** | Explain the scientific background and rationale for the reported study. What is the exposure? Is a potential causal relationship between exposure and outcome plausible? Justify why MR is a helpful method to address the study question | 2 | In youths, we applied a combined MR and twin-based direction-of-causation (MR-DoC) approach to the European twin sample (199 MZ twin pairs, 257 DZ twin pairs) from the Adolescent Brain Cognitive Development (ABCD) Study ®. |
| 3 | **Objectives** | State specific objectives clearly, including pre-specified causal hypotheses (if any). State that MR is a method that, under specific assumptions, intends to estimate causal effects | 4-5 | Oour second aim was to apply MR-DoC to total cortical brain volume (TCBV), TCSA, ACT, and suicide measures from the European (EUR) twin sample from the Adolescent Brain Cognitive Development (ABCD) Study^®^. |
|  | **METHODS** |  |  |  |
| 4 | **Study design and data sources** | Present key elements of the study design early in the article. Consider including a table listing sources of data for all phases of the study. For each data source contributing to the analysis, describe the following: |  |  |
|  | a) | Setting: Describe the study design and the underlying population, if possible. Describe the setting, locations, and relevant dates, including periods of recruitment, exposure, follow-up, and data collection, when available. | 8 | “The ABCD study is a national longitudinal study of adolescent brain development with genetic, brain imaging, and behavioral/psychiatric data from almost 12,000 adolescents aged 9-10 years at baseline across the US22.” |
|  | b) | Participants: Give the eligibility criteria, and the sources and methods of selection of participants. Report the sample size, and whether any power or sample size calculations were carried out prior to the main analysis | 13 | We applied the MR-DoC method to the EUR twin sample only from the ABCD Study® due to the lack of summary statistics from GWASs of brain structure from other ancestry groups required for computing PRSs used in MR-DoC analyses (see Methods). At the baseline timepoint, the ABCD study participants were 9-10 years of age. The ABCD EUR twin sample consisted of around 257 MZ and 199 DZ twin pairs. Around 49% of the twin sample were female and the average age was approximately 10.2 years. |
|  | c) | Describe measurement, quality control and selection of genetic variants | NA | We computed PRS and used them as genetic IVs. |
|  | d) | For each exposure, outcome, and other relevant variables, describe methods of assessment and diagnostic criteria for diseases | 8-9 | The main structural brain imaging measures of interest were total brain volume (TCBV), average cortical thickness (ACT), and total cortical surface area (TCSA). Details of T1 and T2 weighted 3D structural brain image acquisition and processing are described elsewhere^23^. Briefly, brain cortical surface and subcortical segmentation were performed using FreeSurfer v5.3. Cortical regions were parcellated using Destrieux and Desikan atlases. We used the recommended inclusion criteria for T1 weighted structural neuroimaging measures to include samples that passed imaging quality control^24^.  Responses to self-reported suicidal thoughts and behaviors (STBs) questionnaire items were obtained from the Kiddie-Schedule for Affective Disorders and Schizophrenia. Suicidality was coded as an ordinal measure with three levels of increasing suicide liability: (i) no STBs, (ii) suicidal ideation only (past or present passive, active with method, active with intent, active with plan, or non-specific active ideation, but no suicide behaviors), and (iii) suicidal behaviors (past or present preparatory actions towards imminent suicide behaviors and suicide attempt including interrupted or aborted suicide attempt).  For other psychiatric and behavioral measures, we obtained parent-reported measures of their child’s DSM-5 Depression, Internalizing, Externalizing, and Total Problems scores from the Child Behavior Checklist (CBCL) instrument as well as self-reported Behavioral Activation System (BAS) Fun-Seeking Scores. Notably, CBCL measures were zero-inflated. To reduce skewness, 1 was added to all responses (to ensure non-zero values) and then subjected to a log10 transformation, a common approach that may be taken^25^. |
|  | e) | Provide details of ethics committee approval and participant informed consent, if relevant | 25-26 | “This study was approved by the Institutional Review Board at Virginia Commonwealth University which deemed it qualified for exemption (HM20025460) according to 45 CFR 46 category 4 (ii): Secondary research for which consent is not required when information is recorded in such a manner that the identity of the subjects cannot readily be ascertained, the investigator does not contact the subjects, and will not re-identify subjects.” |
| 5 | **Assumptions** | Explicitly state the three core IV assumptions for the main analysis (relevance, independence and exclusion restriction) as well assumptions for any additional or sensitivity analysis | Supplementary Methods | While MR is a powerful tool to probe causality, it relies on several strong assumptions, including that genetic IVs are strongly associated with the exposure of interest (relevance assumption), are not associated with any confounders of the exposure and outcome (independence assumption), and only associated with the outcome through the exposure (the exclusion restriction assumption), and not through alternative mechanisms, which would be considered horizontal pleiotropic associations^3^ |
| 6 | **Statistical methods: main analysis** | Describe statistical methods and statistics used |  |  |
|  | a) | Describe how quantitative variables were handled in the analyses (i.e., scale, units, model) | 9 | “…most CBCL measures were zero-inflated (the majority of responses being 0), so we added 1 to all responses (to ensure all responses were non-zero) and then applied a log10 transformation to reduce skewness. We then residualized all PRS, brain, and psychiatric/behavioral measures by regressing out the first 20 within-ancestry genetic PCs, sex, and interview age as fixed effects as well as study site as a random effect.The distributions of residuals were then visually inspected to approximate normality.” |
|  | b) | Describe how genetic variants were handled in the analyses and, if applicable, how their weights were selected | Supplementary Methods | To compute the PRSs, we used PRS-CS^15^ to generate posterior SNP effect estimates using the QC’d GWAS summary statistics and an external European LD reference panel constructed from the UK Biobank data. We then used PLINK’s (v1.9) --score command to compute individual-level PRSs using the posterior SNP effect estimates and the QC’d target ABCD study imputed genotype data. |
|  | c) | Describe the MR estimator (e.g. two-stage least squares, Wald ratio) and related statistics. Detail the included covariates and, in case of two-sample MR, whether the same covariate set was used for adjustment in the two samples | 9 | For PRSs, as well as brain and psychiatric/behavioral measures of interest, we regressed out the effects of the top 20 within-ancestry genetic principal components (PCs), sex, and interview age as fixed effects. Additionally, we regressed out any confounding effects due to study site as a random effect. The distributions of residuals were then visually inspected to ensure approximate normality. |
|  | d) | Explain how missing data were addressed | NA | NA |
|  | e) | If applicable, indicate how multiple testing was addressed | 10 | The p-values of the causal effect estimates from the MR-DoC models were adjusted for multiple testing by applying Benjamini-Hochberg’s false-discovery rate (FDR) correction^32^. |
| 7 | **Assessment of assumptions** | Describe any methods or prior knowledge used to assess the assumptions or justify their validity | NA | NA |
| 8 | **Sensitivity analyses and additional analyses** | Describe any sensitivity analyses or additional analyses performed (e.g. comparison of effect estimates from different approaches, independent replication, bias analytic techniques, validation of instruments, simulations) | 14-15 | One major assumption of the MR-DoC model is the absence of unique environmental confounding (covE) or factors confounding the exposure and outcome in one twin of a twin-pair, but not in the other twin. To determine how different values of covE would affect the causal estimates, we performed additional sensitivity analyses fixing covE to incrementally smaller and larger values. For all models, larger positive values of covE were associated with more negative causal estimates while more negative covE was associated with more positive causal estimates **(Supplementary Figure 4A-J).** |
| 9 | **Software and pre-registration** |  |  |  |
|  | a) | Name statistical software and package(s), including version and settings used | 10 | The *umx*^28^ and *OpenMx*^29^ R packages were used to fit MR-DoC models to twin data from the ABCD study. Specifically, the *umxMRDoC* function from the *umx* package was used to fit MR-DoC models. |
|  | b) | State whether the study protocol and details were pre-registered (as well as when and where) | NA | This study was not pre-registered. |
|  | **RESULTS** |  |  |  |
| 10 | **Descriptive data** |  |  |  |
|  | a) | Report the numbers of individuals at each stage of included studies and reasons for exclusion. Consider use of a flow diagram | NA | NA for MR-DoC analyses. |
|  | b) | Report summary statistics for phenotypic exposure(s), outcome(s), and other relevant variables (e.g. means, SDs, proportions) | T1 | See Table 1. |
|  | c) | If the data sources include meta-analyses of previous studies, provide the assessments of heterogeneity across these studies | NA | NA for MR-DoC analyses. |
|  | d) | For two-sample MR:  i.  Provide justification of the similarity of the genetic variant-exposure associations between the exposure and outcome samples  ii.  Provide information on the number of individuals who overlap between the exposure and outcome studies | NA | NA for MR-DoC analyses. |
| 11 | **Main results** |  |  |  |
|  | a) | Report the associations between genetic variant and exposure, and between genetic variant and outcome, preferably on an interpretable scale | ST6, ST7 | See Supplementary Tables 6 and 7 |
|  | b) | Report MR estimates of the relationship between exposure and outcome, and the measures of uncertainty from the MR analysis, on an interpretable scale, such as odds ratio or relative risk per SD difference | F3 | See Figure 3 |
|  | c) | If relevant, consider translating estimates of relative risk into absolute risk for a meaningful time period | NA | NA for MR-DoC analyses. |
|  | d) | Consider plots to visualize results (e.g. forest plot, scatterplot of associations between genetic variants and outcome versus between genetic variants and exposure) | F3 | See Figure 3. |
| 12 | **Assessment of assumptions** |  |  |  |
|  | a) | Report the assessment of the validity of the assumptions | Stables 6-7 | See supplementary tables 6 (horizontal pleiotropy) and 7 (instrument strength). |
|  | b) | Report any additional statistics (e.g., assessments of heterogeneity across genetic variants, such as *I^2^*, Q statistic or E-value) | NA | NA for MR-DoC analyses. |
| 13 | **Sensitivity analyses and additional analyses** |  |  |  |
|  | a) | Report any sensitivity analyses to assess the robustness of the main results to violations of the assumptions | 15 | To determine how different values of covE would affect the causal estimates, we performed additional sensitivity analyses fixing covE to incrementally smaller and larger values. For all models, larger positive values of covE were associated with more negative causal estimates while more negative covE was associated with more positive causal estimates **(Supplementary Figure 4A-J).** |
|  | b) | Report results from other sensitivity analyses or additional analyses | 15 | Finally, to assess whether the significant findings in twins of EUR ancestry may generalize to a more diverse population, we applied the hybrid twin-DoC model, which does not require PRSs as genetic IVs, to the full ABCD study twin sample. The full twin sample consisted of around 397 DZ and 308 MZ twin pairs, of which around 33-34% self-identified as non-white. No differences in demographic, brain, or psychiatric and behavioral measures were found between MZ and DZ twins **(Supplementary Table 1)**. We found significant negative causal effects of ACT on depression (-0.24 ± 0.09, p.adj = 0.03) and internalizing (-0.22 ± 0.09, p.adj = 0.03) psychopathology, as well as significant negative reverse causal effects of depression (-0.10 ± 0.04, p.adj = 0.03) and internalizing (-0.09 ± 0.04, p.adj = 0.03) on ACT **(Figure 3C).** |
|  | c) | Report any assessment of direction of causal relationship (e.g., bidirectional MR) | 14 | For our second set of MR-DoC analyses, we tested the reverse causal hypotheses that suicidality and associated psychiatric/behavioral risk factor measures causally influences brain structure. We did not find any significant causal effects of suicidality on any of the brain measures. However, we did find significant negative causal effects of depression on TCBV (-0.07 ± 0.03, p.adj = 0.03) and ACT (-0.13 ± 0.04 p.adj = 0.03), of internalizing on TCBV (-0.07 ± 0.02, p.adj = 0.03) and ACT (-0.10 ± 0.04, p.adj = 0.04), and of total problems on TCBV (-0.09 ± 0.03, p.adj =0.03) **(Figure 3B).** |
|  | d) | When relevant, report and compare with estimates from non-MR analyses | NA | NA for MR-DoC analyses. |
|  | e) | Consider additional plots to visualize results (e.g., leave-one-out analyses) | NA | NA for MR-DoC analyses. |
|  | **DISCUSSION** |  |  |  |
| 14 | **Key results** | Summarize key results with reference to study objectives | 16  17 | In youths of EUR ancestry, we did not find a significant causal effect of lower TCBV, TCSA, or ACT on increased suicidality risk or any reverse causal effects.  However, we did find evidence that lower TCBV may cause increased depression in youths of EUR ancestry. However, these effects were not specific as TCBV was also shown to exhibit significant negative causal influences on internalizing psychopathology and total problem scores. |
| 15 | **Limitations** | Discuss limitations of the study, taking into account the validity of the IV assumptions, other sources of potential bias, and imprecision. Discuss both direction and magnitude of any potential bias and any efforts to address them | 18 | Finally, we acknowledge several limitations to the approaches we used in this study. Due to our focus on global rather than regional brain structure measures, we were unable to identify specific functional brain circuits and mechanisms related to suicide risk. Additionally, we did not adjust our brain and behavioral measures for the potential effects of medications in our MR-DoC analyses. As well, while we did adjust brain and behavioral measures by sex, we did not characterize any sex differences in our findings. |
| 16 | **Interpretation** |  |  |  |
|  | a) | Meaning: Give a cautious overall interpretation of results in the context of their limitations and in comparison with other studies | 18 | Altogether, our findings suggest that suicide risk may be instantiated differently in adults compared to youth. |
|  | b) | Mechanism: Discuss underlying biological mechanisms that could drive a potential causal relationship between the investigated exposure and the outcome, and whether the gene-environment equivalence assumption is reasonable. Use causal language carefully, clarifying that IV estimates may provide causal effects only under certain assumptions | 16 | One interpretation might be that decreases in ACT may underlie increased suicide capability, which may result from prior suicide attempts, or other painful and provocative events^3^. Indeed, one study found significantly thinner cortices in the left dorsolateral, ventromedial prefrontal and anterior cingulate cortices in patients at high risk for suicide compared to non-high-risk patients^35^. |
|  | c) | Clinical relevance: Discuss whether the results have clinical or public policy relevance, and to what extent they inform effect sizes of possible interventions | 18 | Altogether, our findings suggest that suicide risk may be instantiated differently in adults compared to youth. As such, clinical interventions aimed at reducing the rate by which cortical surface area decreases in later adolescence may more effectively decrease the risk of suicide in adults. Contrastingly, interventions targeting average cortical thickness and depressive/internalizing psychopathology in earlier in adolescence or childhood may be more beneficial in reducing future suicide risk. |
| 17 | **Generalizability** | Discuss the generalizability of the study results (a) to other populations, (b) across other exposure periods/timings, and (c) across other levels of exposure | 17 | “Importantly, these effects were also found using the traditional twin-DoC method in the full ABCD twin sample. Since about one third of the full twin sample includes individuals of non-white race/ethnicity, these findings may be more generalizable across diverse youth populations.” |
|  | **OTHER INFORMATION** |  |  |  |
| 18 | **Funding** | Describe sources of funding and the role of funders in the present study and, if applicable, sources of funding for the databases and original study or studies on which the present study is based | Title Page |  |
| 19 | **Data and data sharing** | Provide the data used to perform all analyses or report where and how the data can be accessed, and reference these sources in the article. Provide the statistical code needed to reproduce the results in the article, or report whether the code is publicly accessible and if so, where | 26-27 | “GWAS summary statistics for total brain cortical surface area9 and suicide attempt10 were obtained from publicly available repositories.  Data used in the preparation of this article were obtained from the Adolescent Brain Cognitive DevelopmentSM (ABCD) Study (https://abcdstudy.org), held in the NIMH Data Archive (NDA)…”  “The scripts used for genetic ancestry assignment and genetic principal component analysis are available upon request. Scripts for all the other analyses supporting this study are available at our Open Science Framework repository for this study: https://osf.io/xe8yp/?view_only=3daf23ae35e94c1d92d8d31326ca4a1d” |
| 20 | **Conflicts of Interest** | All authors should declare all potential conflicts of interest | 27 | “The author(s) declare no competing interests.” |

This checklist is copyrighted by the Equator Network under the Creative Commons Attribution 3.0 Unported (CC BY 3.0) license.

1. Skrivankova VW, Richmond RC, Woolf BAR, Yarmolinsky J, Davies NM, Swanson SA, et al. Strengthening the Reporting of Observational Studies in Epidemiology using Mendelian Randomization (STROBE-MR) Statement. JAMA. 2021;under review.

2. Skrivankova VW, Richmond RC, Woolf BAR, Davies NM, Swanson SA, VanderWeele TJ, et al. Strengthening the Reporting of Observational Studies in Epidemiology using Mendelian Randomisation (STROBE-MR): Explanation and Elaboration. BMJ. 2021;375:n2233.
