# Supplementary material for: Causal Analyses of Associations Between Brain Structure and Suicide Attempt in Adulthood and Late Childhood
Source: JAACAP Open. 2025 Mar 21;3(3):455–66. doi: 10.1016/j.jaacop.2025.02.005 (PMC12414325; doi:10.1016/j.jaacop.2025.02.005)
Supplement: Supplementary Material [file mmc4.docx]

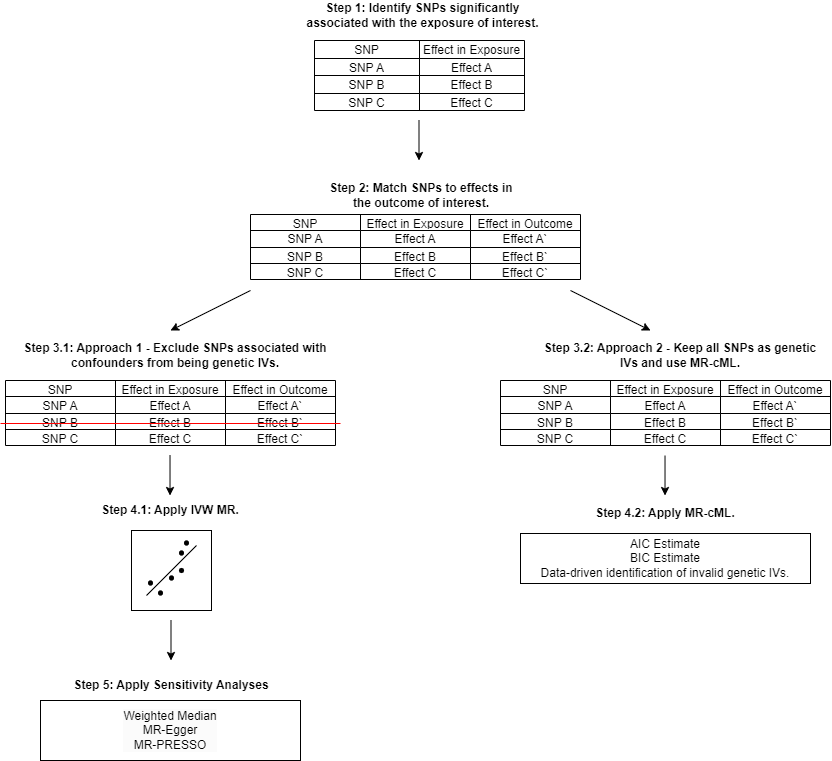


**Figure S1 – Two-Sample Mendelian Randomization (MR) approach.** Note: In step 1, plausible genetic instrumental variables (IV) were first selected by finding Single Nucleotide Polymorphisms (SNPs) significantly associated with the exposure of interest. Importantly, only linkage disequilibrium (LD)-independent SNPs (such as those identified by using PLINK^20^ to perform LD pruning with the following parameters: LD R^2^ = 0.1, 250k base-pair windows) were selected. In step 2, matching SNPs with corresponding association estimates for the outcome measure were identified. In step 3.1, a more conservative approach where SNPs associated with potential confounding factors were excluded as genetic IVs in subsequent MR analyses (steps 4.1 and 5). In Step 3.2, all SNPs were included as genetic IVs and a data driven approach was used to identify invalid IVs and estimate the causal effect simultaneously (Step 4.2).

**A) B)**


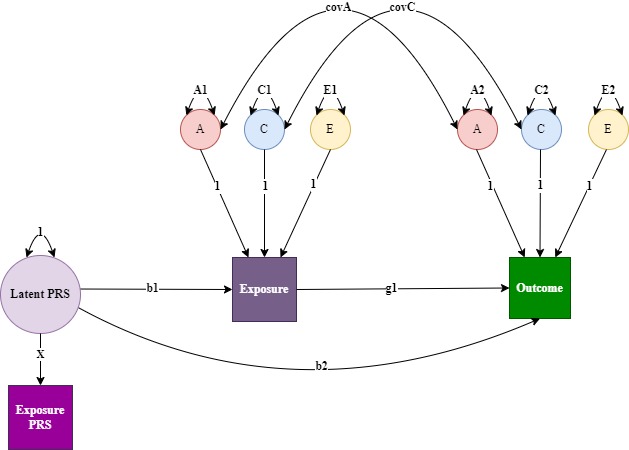

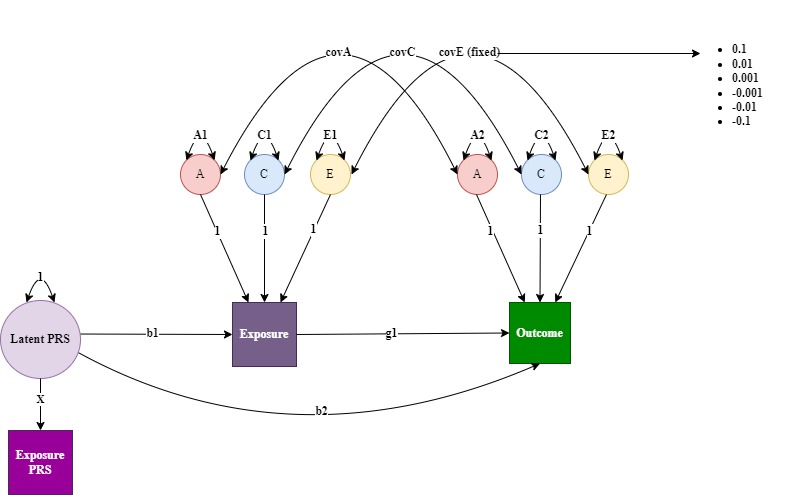


**Figure S2 – A) Path Diagram for the main Mendelian Randomization Direction of Causation (MR-DoC) model in one twin of a twin-pair.** Note: A, C, and E represent latent additive genetic, shared environmental, and unique environmental variance components of the exposure and outcome measures. Additive genetic, shared environmental, and unique environmental confounding are represented by covA, covC, and covE respectively. A polygenic risk score (PRS) is used as a genetic instrumental variable (IV) for the exposure of interest. The causal effect of the exposure on the outcome, instrumental variable-exposure association, and horizontal pleiotropic associations are represented by g1, b1, and b2, respectively. The standard deviation of observed PRS is represented by x. Single-headed arrows represent linear regressions, while double-headed arrows between and within variables represent covariances and variances, respectively. **B)** Path diagram for sensitivity analyses with different fixed values of covE to assess the impact on the causal estimate, g1.

**A) B)**

**
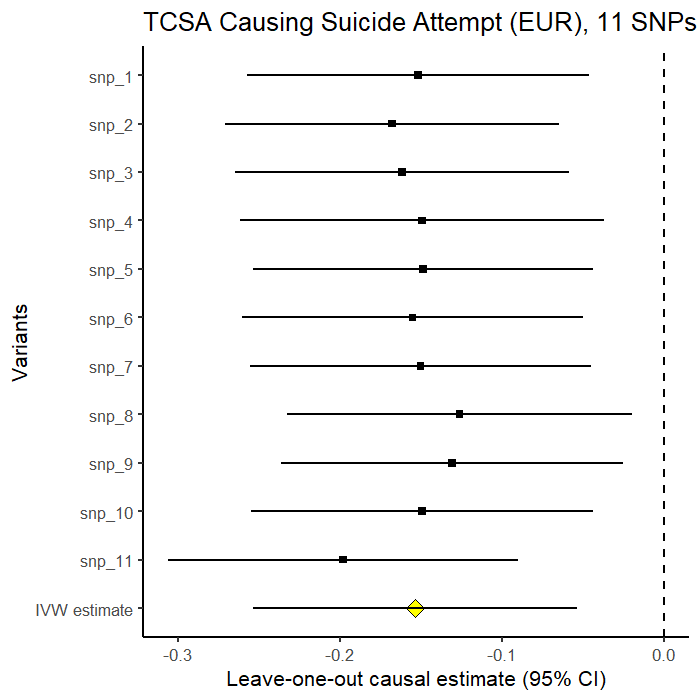
** **
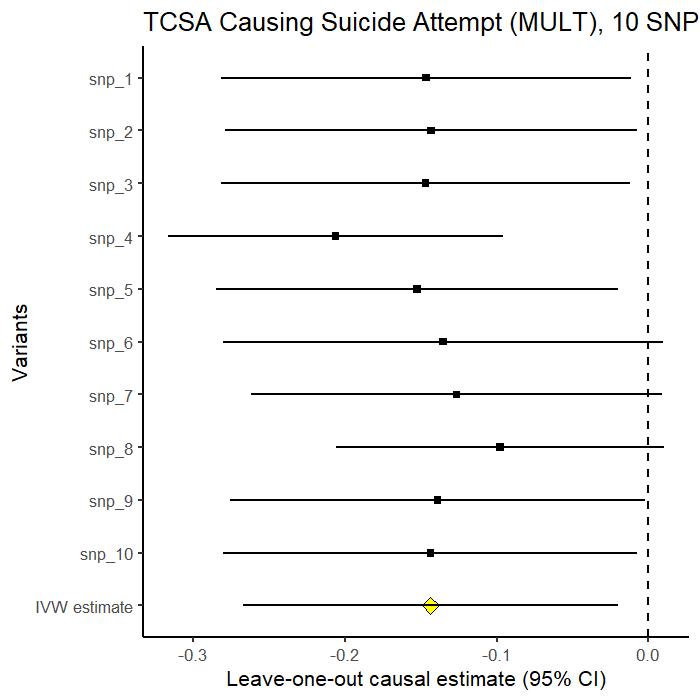
**

**Figure S3 – Leave-One-Out analyses for Inverse-Variance-Weighted causal estimates for Total Cortical Surface Area (TCSA) on suicide attempt using summary statistics from GWAS of suicide attempt from A) European ancestry or B) Multi-Ancestry adult samples.**


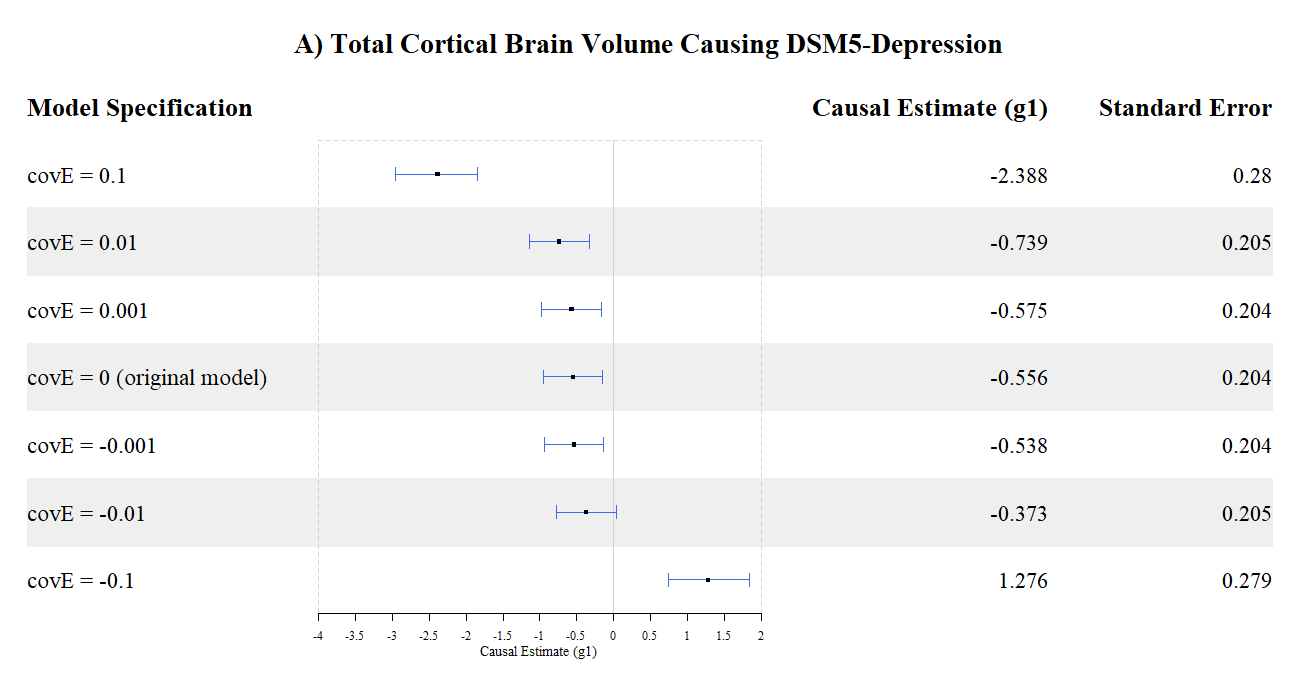

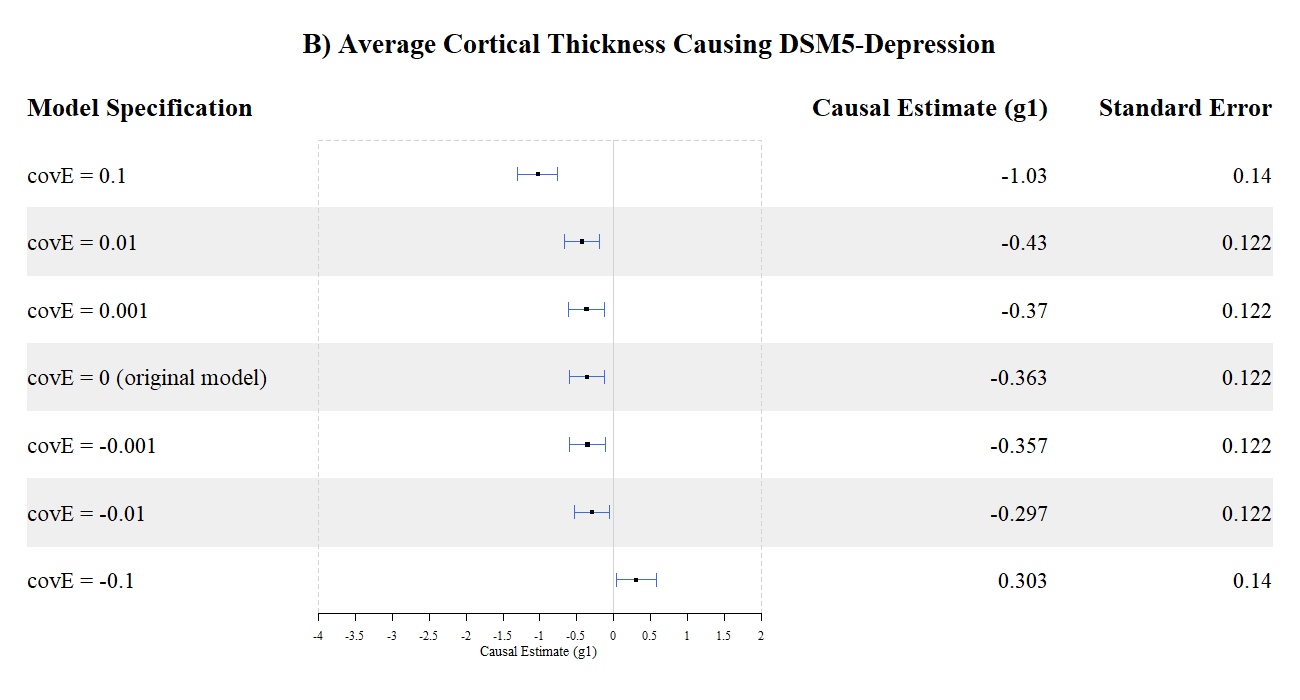


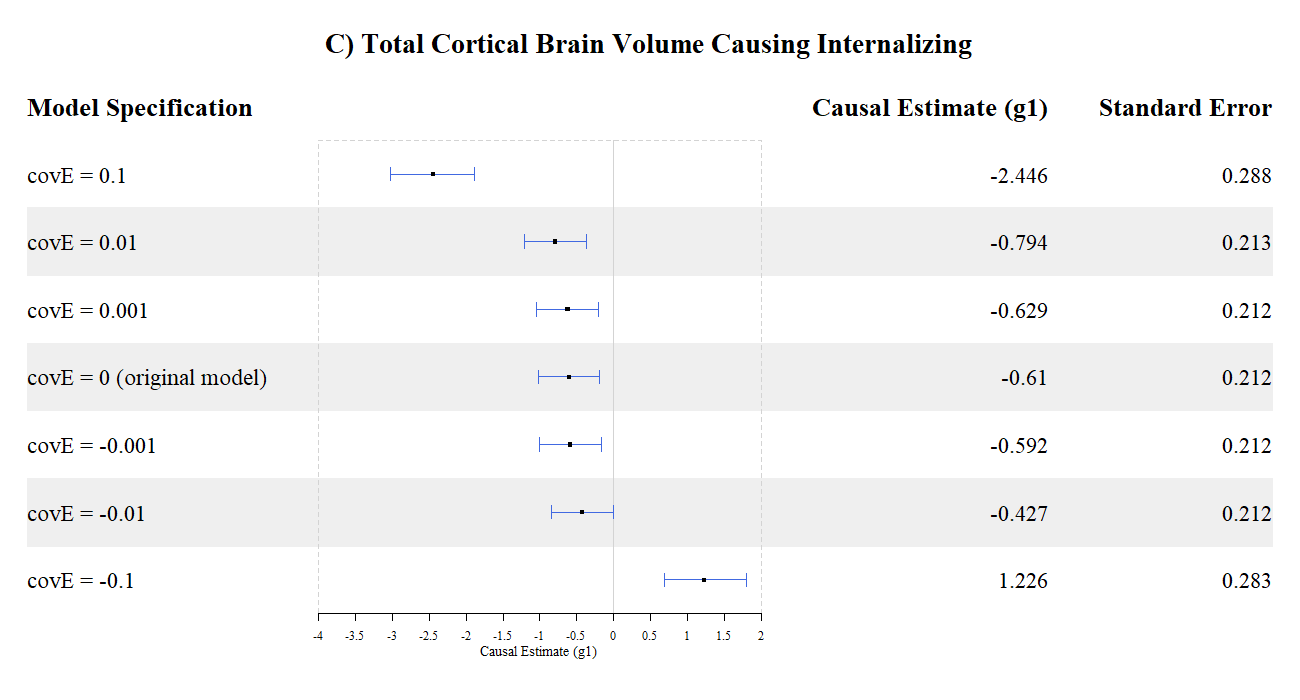

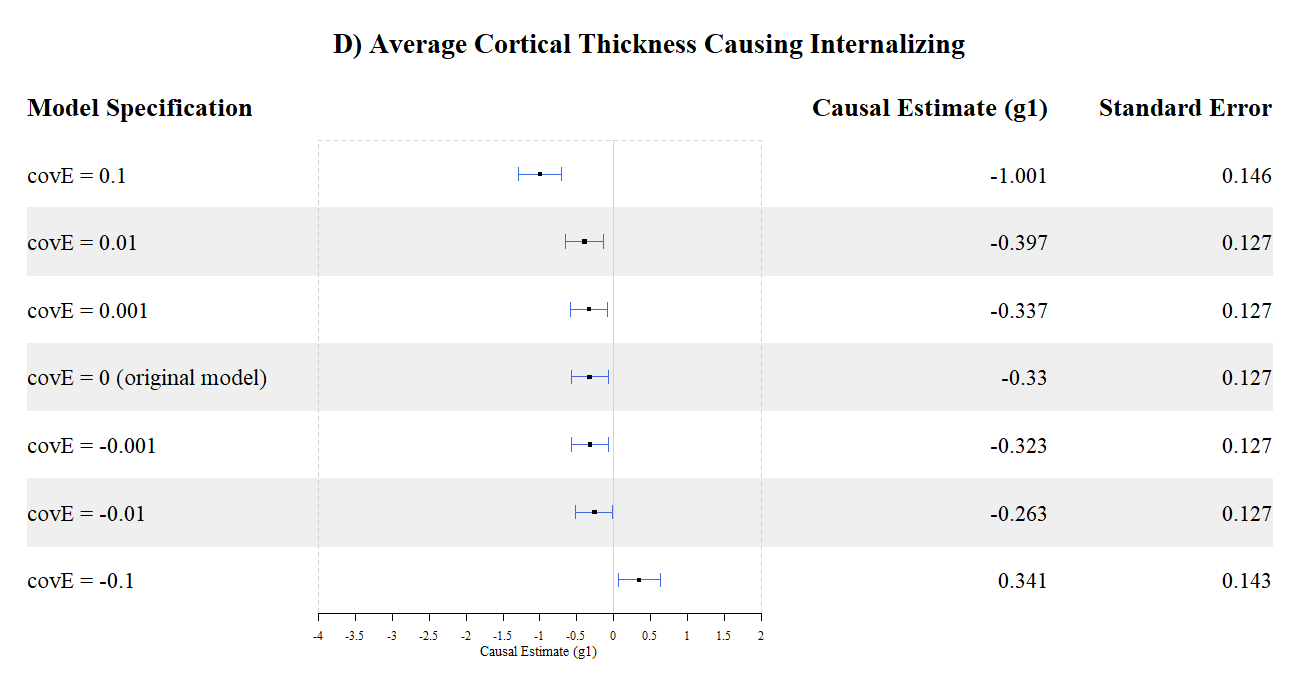


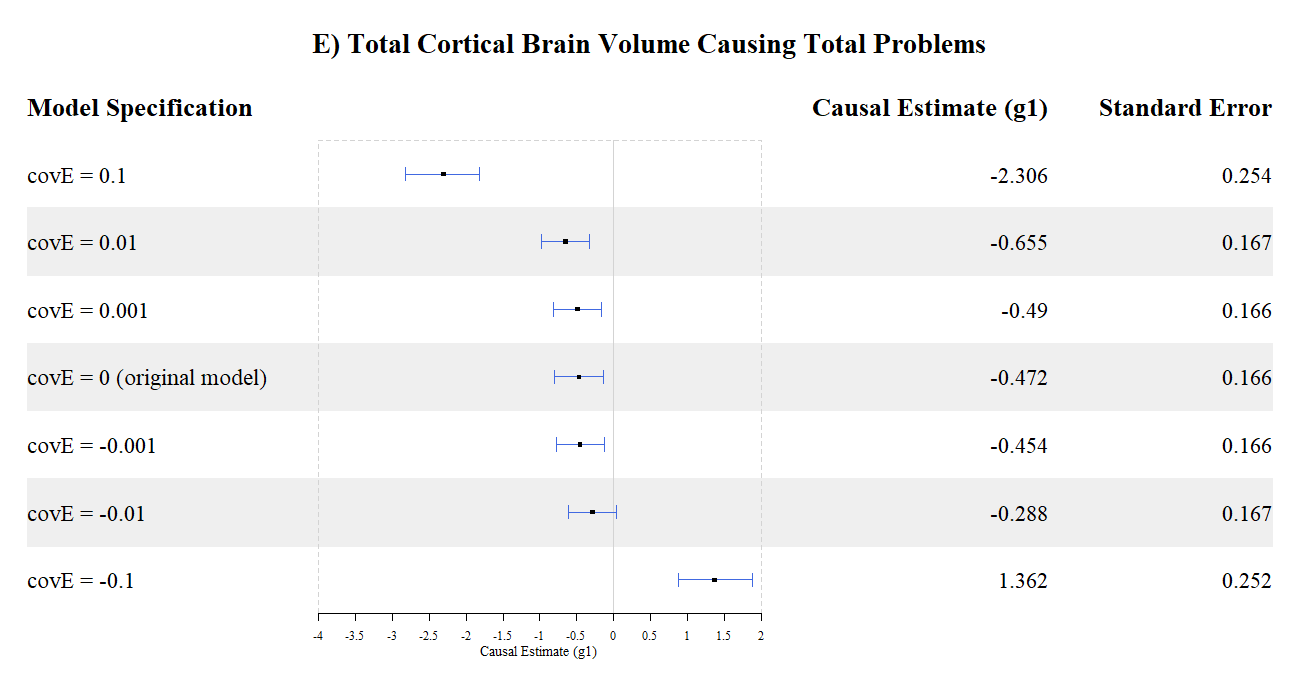

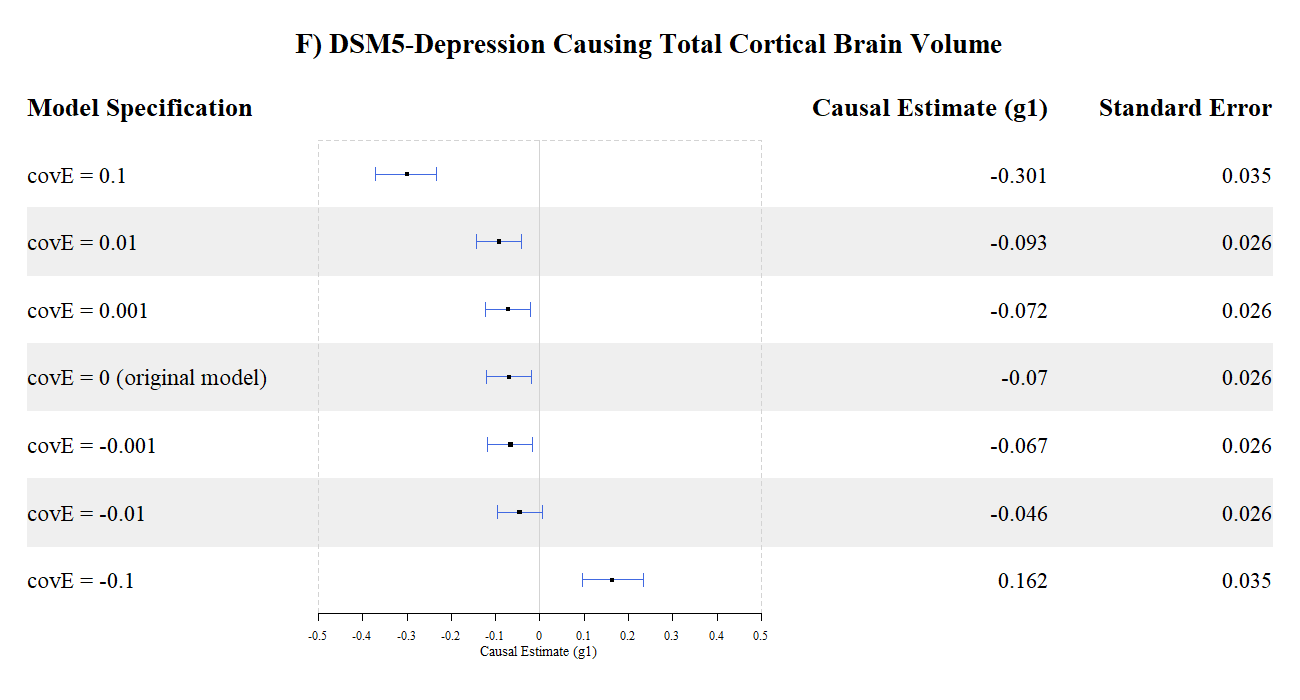


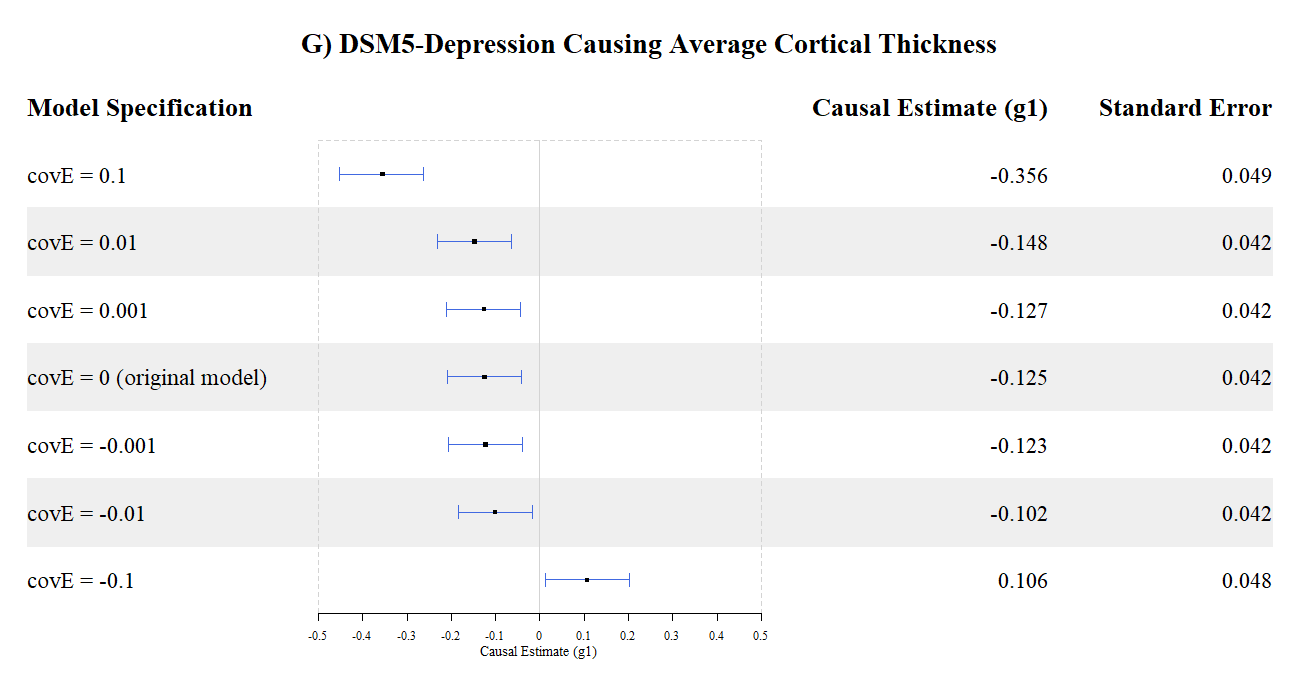

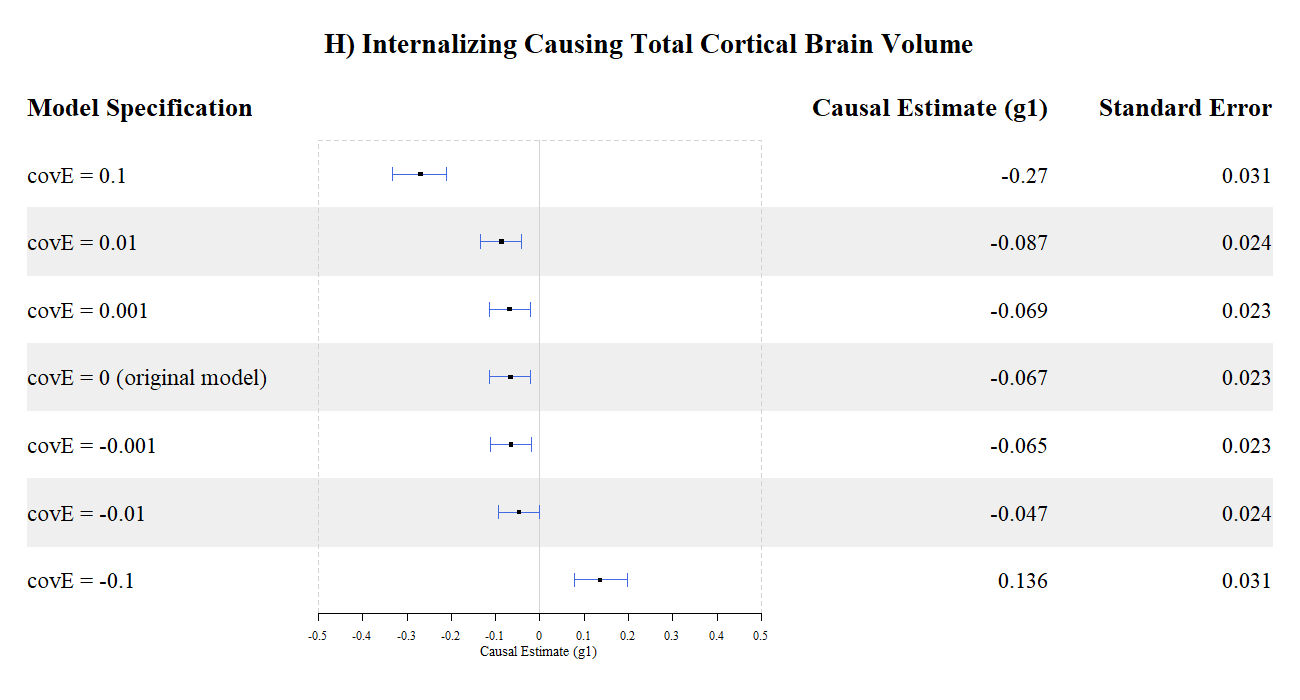


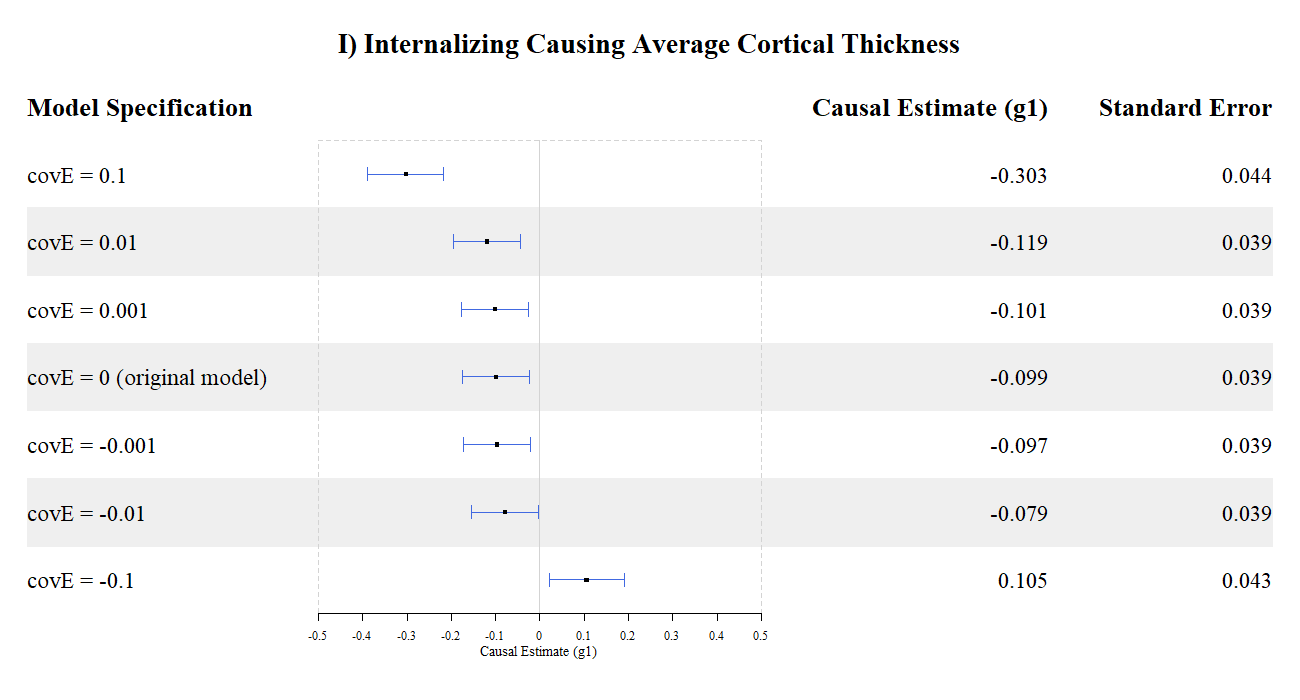

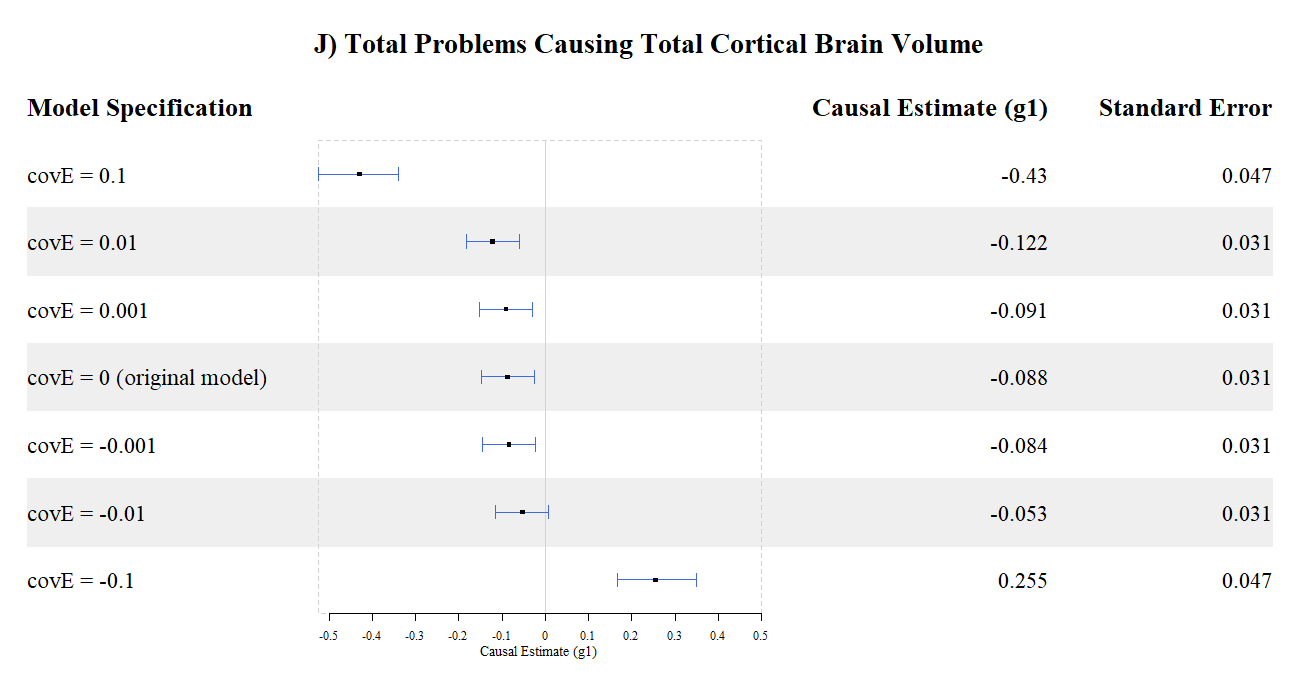


**Figure S4 – MR-DoC sensitivity analyses.** Note: Here, the values for unique environmental confounding (covE) effects are varied from -0.1 to 0.1 and the impact on the causal estimates (g1) are observed. Error bars represent 95% confidence intervals.
